# Clinical characteristics and variant spectrum of *NF1* in Chinese patients with neurofibromatosis type 1: a cross-sectional study

**DOI:** 10.1186/s13023-026-04534-9

**Published:** 2026-08-17

**Authors:** Ya-Xin Guo, Xin-De Liu, Yi-Qiu Yan, He-Xiao Wang, Xiao-Shi Yang, Yuan Lv, Gang Ma, David Croitoru, Rui-Qun Qi, Yan Wu, Ting Xiao, Long Geng, Vincent Piguet, Hong-Duo Chen, Eric Legius, Xue-Gang Xu, Xing-Hua Gao

**Affiliations:** 1 Department of Dermatology, The First Hospital of China Medical University, No.155 Nanjing Bei Street, Heping District, Shenyang, Liaoning Province 110001 China; 2 Key Laboratory of Immunodermatology, Ministry of Education and NHC, National joint Engineering Research Center for Theranostics of Immunological Skin Diseases, No.155 Nanjing Bei Street, Heping District,, Shenyang, Liaoning Province 110001 China; 3 Department of General Surgery, The Fourth Affiliated Hospital of China Medical University, Shenyang, China; 4 Department of Social Medicine, College of Health Management, China Medical University, Shenyang, China; 5 Department of Gynecology and Obstetrics, Shenyang Women and Children’s Hospital, Shenyang, China; 6 Bio-X Institutes, Key Laboratory for the Genetics of Developmental and Neuropsychiatric Disorders, Shanghai Jiao Tong University, Ministry of Education, Shanghai, China; 7 Division of Dermatology, Department of Medicine, University of Toronto, Toronto, ON Canada; 8 Division of Dermatology, Women’s College Hospital, Toronto, ON Canada; 9 Department of Human Genetics, KU Leuven, Leuven, Belgium; 10 Department of Clinical Genetics, UZ Leuven, Leuven, Belgium; 11 Full Member of the European Reference Network on Genetic Tumour Risk Syndromes (ERN GENTURIS), Nijmegen, The Netherlands

**Keywords:** Neurofibromatosis type 1, *NF1* variant spectrum, Genotype-phenotype relationships, Whole-exome sequencing, Cross-sectional study

## Abstract

**Background:**

Neurofibromatosis type 1 (NF1) is a rare autosomal dominant multisystem disorder caused by *NF1* gene variants. Although NF1 shows marked clinical and genetic heterogeneity, large Chinese cohorts integrating clinical features, *NF1* variant spectrum, and external variant contextualization remain limited.

**Methods:**

We conducted a cross-sectional study of 847 clinically confirmed Chinese patients with NF1 to characterize demographic features, clinical manifestations, DNB-defined severity, and *NF1* variant spectrum. Whole-exome sequencing was performed in 211 patients. Transcript-level variant distribution was assessed using a 500-bp sliding-window approach and further contextualized using ClinVar-derived *NF1* variant data.

**Results:**

Among 847 patients, the median age was 23 years, 27.5% had a family history of NF1, and more than 90% were younger than 40 years. Café-au-lait macules, neurofibromas, and plexiform neurofibromas were observed in 98.3%, 70.7%, and 20.9% of patients, respectively. Younger age, lower neurofibroma burden, and absence of learning difficulties were associated with DNB-defined mild classification. Among 211 genetically tested patients, pathogenic/likely pathogenic *NF1* variants were identified in 182 patients, 11 carried *NF1* variants of uncertain significance, and 18 had no reportable *NF1* variant. In total, 152 distinct *NF1* variants were identified, including 45 novel sites. Transcript-level analysis showed regional variation in reportable nucleotide-level *NF1* variants, with relatively higher variant density around exons 10–15 and 44–49. Comparison with ClinVar pathogenic/likely pathogenic *NF1* variants showed no significant window-level difference after multiple-testing correction.

**Conclusions:**

This cross-sectional study summarizes the clinical and genetic features of Chinese patients with NF1 and expands the known *NF1* variant spectrum in this population. Transcript-level analysis showed regional variation in reportable *NF1* variants, broadly paralleling the ClinVar pathogenic/likely pathogenic *NF1* variant distribution. These findings provide a basis for future longitudinal studies to validate clinically meaningful genotype–phenotype relationships in NF1.

**Supplementary Information:**

The online version contains supplementary material available at https://doi.org/10.1186/s13023-026-04534-9.

## Background

Neurofibromatosis type 1 (NF1, OMIM 162200), previously referred to as von Recklinghausen neurofibromatosis, is a rare autosomal dominant multisystem disorder affecting approximately 1 per 2500 to 3000 individuals worldwide [1–4]. A positive family history is reported in approximately half of the NF1 patients [5–7]. Clinical manifestations of NF1 include multiple café-au-lait macules (CALMs), neurofibromas, axillary and/or inguinal freckles, Lisch nodules, bony abnormalities, learning disabilities and epilepsy, etc. NF1 patients are also at increased risk of developing benign and malignant tumors [8–10], and normally have reduced quality of life [11–14]. Life expectancy of NF1 patients may be reduced by 8 to 21 years when compared to the healthy population [15–17]. The mechanisms determining the variable clinical features of NF1 patients are rather complex and poorly understood. Even in the same family, members with NF1 could present variable and unpredictable clinical features, highlighting the complex pathobiology of mutations in neurofibromin [18, 19]. 

Genetic variants in the tumor suppressor gene of *NF1* are widely accepted as the leading cause of this disease. This gene occupies approximately 350 kb on chromosome 17q11.2 that contains 57 constitutive exons and three alternatively spliced exons [20–22]. Alterations in *NF1* lead to downregulation or inactivation of neurofibromin, a Ras GTPase-activating protein consisting of 2818 amino acids, and activation of RAS/RAF/MEK/ERK and PI3K/Akt/mTOR signaling pathways, with ultimate result of excessive cell proliferation and multisystem tumor formation [23–25]. MEK inhibitors have recently demonstrated significant effectiveness in clinical trials for treating plexiform neurofibromas (PN), leading to high partial response rates and the Food and Drug Administration’s approval of selumetinib in 2020 for pediatric patients [26–28]. 

Although more than 2000 pathogenic variants of *NF1* are included in public databases such as the Human Gene Mutation Database (HGMD) (http://www.hgmd.org) and ClinVar from NCBI, no mutational hotspots have been identified [8]. The exact correlations between genotype and phenotype in NF1 patients remain unclear. Thus, the objective of this study is to establish a large-scale cohort of Chinese NF1 patients in order to analyze its demographics, clinical characteristics, and spectrum of *NF1* variants, as well as to explore the genotype-phenotype relationships in depth, which is crucial for prenatal counseling, disease prognostication, surveillance, and eventually establishment of better clinical management.

## Methods

### Patients

A cross-sectional survey was conducted from September 2017 to May 2022. This survey included 847 patients with clinically confirmed NF1 recruited from the outpatient clinics of the First Hospital of China Medical University and through the Shenzhen Bubble Home Neurofibromatosis Care Center, Guangdong Province, China [29]. Bubble Home is a nonprofit patient advocacy and care organization for individuals with neurofibromatosis and their families in China. In this study, Bubble Home served as a nationwide patient outreach and referral platform by disseminating study information and assisting potentially eligible patients and families in contacting the study investigators. It did not independently diagnose NF1 or determine eligibility for study inclusion. Patients meeting the diagnostic criteria for NF1 established by the National Institutes of Health in 1988 were included in the study [30]. For patients recruited from the outpatient clinics of the First Hospital of China Medical University, the diagnosis of NF1 was made directly by clinicians in the study team. For patients referred through the Neurofibromatosis Care Center, the initial diagnosis was generally made by local physicians and was subsequently reviewed by the study team using available clinical records, photographs, examination findings, and/or genetic testing results. Final eligibility for inclusion was determined by the study team. Diagnoses were reviewed by at least two experienced dermatologists/genetic clinicians, and discrepant cases were adjudicated by senior investigators. Patients who declined or were unable to provide informed consent, or whose essential diagnostic information was insufficient to confirm NF1 according to the 1988 NIH criteria, were excluded. No additional exclusion criteria were applied on the basis of disease severity, malignancy history, cognitive impairment, or treatment history, because these conditions may constitute part of the clinical spectrum or complications of NF1. For children or patients unable to complete the questionnaire independently, information was provided by parents or legal guardians and cross-checked with available medical records, photographs, examination findings, and/or genetic testing results when available. Incomplete phenotype data were coded as missing rather than assumed to be absent. The study was approved by the Ethics Committee of the First Hospital of China Medical University (2017-112-2).

### Clinical severity assessment

An in-depth questionnaire (Supplement 1) was used to obtain detailed clinical information of NF1 patients. The severity of NF1 patients was defined according to the Japanese DNB classification criteria (Supplement 2) proposed in 2017; [31, 32] stage 1 was defined as mild type and stages 2–5 as severe type. The DNB system evaluates dermatological, neurological, and bone manifestations and their impact on daily life and social activity. Because NF1 manifestations are age-dependent and DNB staging incorporates clinical features that may also be analyzed as covariates, analyses based on DNB-defined severity were used to assess cross-sectional associations rather than prognostic or causal effects.

### Genetic analysis

EDTA-treated peripheral blood was collected from 211 patients who provided written informed consent for genomic DNA extraction and whole-exome sequencing (WES) [33]. 

The nomenclature of sequence variants follows the guidelines proposed by the Human Genome Variation Society. Variants were classified according to the American College of Medical Genetics and Genomics/Association for Molecular Pathology (ACMG/AMP) guidelines and reported as pathogenic/likely pathogenic, variants of uncertain significance (VUS), or negative. Novelty was determined by cross-referencing ClinVar, HGMD, and Franklin. *NF1* variants were assigned to five predefined variant domains (VDs) where applicable, based on the relationship between protein domains and biochemical functions. Variants spanning more than one predefined VD or not assignable to a single VD were coded as an overlapping/multiple-domain category for exploratory statistical analyses [34]. Genetic variation characteristics of *NF1* include nucleotide change, amino acid change, variant types (nonsense, missense, frameshift, exon deletions, splicing, duplications, synonymous, indel, etc.), variant location (involved exons or introns), alterations in neurofibromin structure (missense variant related alterations were defined as small and other variation type related alterations were defined as big), and Sanger validation (*n* = 92). For transcript-level distribution analysis, nucleotide-level *NF1* variants were mapped to cDNA coordinates according to NM_000267.4. Exon-level deletions and duplications were excluded from this density analysis because their breakpoints could not be assigned to a single nucleotide-level position. Variant density was first assessed using a 500-bp sliding window with a 50-bp step across the *NF1* cDNA sequence. To provide external contextualization, we also re-analyzed publicly available *NF1* variants in ClinVar. After excluding intronic and untranslated region (UTR) variants, ClinVar variants were categorized as pathogenic/likely pathogenic, benign/likely benign, or variants of uncertain significance. ClinVar variants were summarized in 16 non-overlapping 500-bp windows. Chi-square goodness-of-fit tests were used to assess deviation from a uniform distribution across windows, and Fisher’s exact tests were used to compare window-level distributions between the present cohort and ClinVar pathogenic/likely pathogenic variants. Benjamini-Hochberg false-discovery rate (FDR) correction was applied for multiple testing.

### Statistical analysis

SPSS version 23.0 statistical software (IBM Corporation) was used for statistical analysis. Descriptive statistics were applied to summarize demographic and clinical data. Rates and composition ratios were used to describe categorical variables. The chi-square test was conducted to determine the relationship between clinical severity and other variables, and to evaluate the differences in distribution between two categories. Multivariable logistic regression analysis was performed for exploring the factors associated with DNB-defined severity, along with gender, age, etc., to control for potential confounding factors. Odds ratios (OR) and 95% confidence intervals (CI) were calculated when a significant effect was observed. Where multiple comparisons were performed, FDR correction was applied to account for multiplicity, with q < 0.05 considered statistically significant. Missing data were coded as missing and were not imputed as absent.

## Results

### Demographic characteristics

In total, 847 NF1 patients with a median age of 23 (range: 1 month to 70 years) were included in this study, in which 27.5% have family history. The male to female ratio was 359 to 488 (approximately 3:4). Notably more than 90% of the patients were aged younger than 40 years. Of all age groups, patients aged < 10 years constituted the largest age subgroup (35.7%) (Supplement 3). The percentage of patients at DNB stage 1 decreased from 77.5% in pediatric patients (< 10 years) to 9.5% in patients aged between 40 and 49, and patients aged between 50 and 59 showed the highest proportion of DNB stage 5 (16.7%) (Table 1).

**Table 1:** Age-stratified distribution of DNB stages among 847 patients with NF1

Age group	Stage 1 (%)	Stage 2 (%)	Stage 3 (%)	Stage 4 (%)	Stage 5 (%)	Total
0 ~ 9	234 (77.5)	51 (16.9)	1 (0.3)	0 (0)	16 (5.3)	302
10 ~ 19	52 (55.3)	33 (35.1)	0 (0)	1 (1.1)	8 (8.5)	94
20 ~ 29	65 (29.5)	137 (62.3)	3 (1.4)	2 (0.9)	13 (5.9)	220
30 ~ 39	30 (18.1)	118 (71.1)	7 (4.2)	4 (2.4)	7 (4.2)	166
40 ~ 49	4 (9.5)	34 (80.9)	0 (0)	2 (4.8)	2 (4.8)	42
50 ~ 59	3 (16.7)	9 (50)	2 (11.1)	1 (5.5)	3 (16.7)	18
≥ 60	0 (0)	3 (60)	1 (20)	0 (0)	1 (20)	5
Total	388 (45.8)	385 (45.4)	14 (1.7)	10 (1.2)	50 (5.9)	847

Values are presented as n (%), with percentages calculated within each age group. DNB stage 1 was classified as mild, and DNB stages 2–5 were classified as severe. Percentages may not total 100% because of rounding

### Clinical characteristics

Demographic and clinical characteristics were indicated in Table 2. Most cases presented with CALMs (98.3%) and neurofibromas (70.7%); neurofibromas were predominant amongst adults (94.6%; 452/478) but in the minority of pre-pubertal children (35.1%; 118/336). The overall proportion of PN was 20.9%. The proportion of patients with skeletal abnormalities and learning difficulties were 17.9% and 16.8%, respectively. Overall, 91.2% of the patients were staged 1–2 under the evaluation by DNB classification criteria. After Benjamini-Hochberg FDR correction, several clinical variables were associated with DNB-defined severity; these included age of onset, course of disease, family history, maximum diameter and quantity of CALMs, presence of CALMs on face, age of onset of neurofibromas, presence of neurofibromas on face, learning disabilities, nevus anemicus (AN) and halo-like phenomenon on dermal melanocytosis.

**Table 2:** Characteristics and clinical severity of 847 NF1 patients

Variables	Total (%) [*n*, (%)]	Clinical severity				
		Mild type (*n*)	Severe type (*n*)	χ²	*P*	q
**Demographic characteristics**						
**Patient origin**				8.485	0.004	0.008
Outpatient clinics	80 (9.4)	49	31			
The Neurofibromatosis Care Center	767 (90.6)	339	428			
**Gender**				1.109	0.292	0.3504
Male	359 (42.4)	172	187			
Female	488 (57.6)	216	272			
**Ethnicity**				0.017	0.896	0.896
Han	784 (92.6)	358	426			
Non-Han minorities	58 (6.8)	27	31			
Missing value	5 (0.6)					
**Age (years)**				228.552	< 0.001	0.0024
≤ 9	302 (35.7)	234	68			
10–25	208 (24.6)	94	114			
> 25	337 (39.8)	60	277			
**Age of onset (years)**				18.502	< 0.001	0.0024
= 0	586 (69.2)	283	303			
> 0–2	133 (15.7)	69	64			
> 2	126 (14.9)	36	90			
Missing value	2 (0.2)					
**Disease course (years)**				213.563	< 0.001	0.0024
≤ 8	285 (33.6)	222	63			
9–25	270 (31.9)	116	154			
> 25	289 (34.1)	50	239			
Missing value	3 (0.4)					
**Family history**				5.904	0.015	0.024
No	614 (72.5)	297	317			
Yes	233 (27.5)	91	142			
**CALMs characteristics**						
**Facial skin involvement at onset**				0.727	0.394	0.4503
No	646 (77.6)	295	351			
Yes	187 (22.4)	92	95			
**Increased number**				3.344	0.067	0.0846
No	94 (11.3)	52	42			
Yes	739 (88.7)	335	404			
**Maximum diameter (cm)**				24.755	< 0.001	0.0024
≤ 5	410 (48.4)	219	191			
6–8	184 (21.7)	84	100			
> 8	253 (29.9)	85	168			
**Quantity**				6.272	0.043	0.0607
0–9	111 (13.1)	57	54			
10–99	508 (60.0)	242	266			
> 99	228 (26.9)	89	139			
**Facial presence currently**				5.329	0.021	0.0315
No	466 (55.9)	200	266			
Yes	367 (44.1)	187	180			
**Neurofibromas characteristics**						
**Age of onset (years)**				9.520	0.009	0.0154
≤ 4	218 (36.4)	79	139			
5–11	178 (29.7)	47	131			
> 11	203 (33.9)	47	156			
**Face involvement at disease onset** (years)				0.045	0.831	0.887
No	471 (78.6)	137	334			
Yes	128 (21.4)	36	92			
**PN**				50.949	< 0.001	0.0024
No	670 (79.1)	349	321			
Yes	177 (20.9)	39	138			
**Quantity**				294.171	< 0.001	0.0024
0	248 (29.3)	215	33			
1–9	210 (24.8)	99	111			
10–99	201 (23.7)	57	144			
> 99	188 (22.2)	17	171			
**Facial presence currently**				7.393	0.007	0.0129
No	379 (63.3)	124	255			
Yes	220 (36.7)	49	171			
**Abnormal skin sensations**				58.477	< 0.001	0.0024
No	398 (66.4)	155	243			
Yes	201 (33.6)	18	183			
**Other characteristics**						
**Axillary and/or inguinal freckles**				0.036	0.850	0.887
No	378 (44.6)	173	205			
Yes	461 (54.4)	214	247			
Missing value	8 (0.9)					
**Bony abnormalities**				79.552	< 0.001	0.0024
No	695 (82.1)	368	327			
Yes	152 (17.9)	20	132			
**Learning disabilities**				26.813	< 0.001	0.0024
No	705 (83.2)	351	354			
Yes	142 (16.8)	37	105			
**AN**				17.960	< 0.001	0.0024
No	676 (79.8)	285	391			
Yes	171 (20.2)	103	68			
**JXGs**				3.640	0.056	0.0747
No	709 (83.7)	335	374			
Yes	138 (16.3)	53	85			
**Halo-like phenomenon on dermal melanocytosis**				8.789	0.003	0.0065
No	661 (78.0)	285	376			
Yes	186 (22.0)	103	83			

Percentages were calculated among patients with available data for each variable. Missing values were excluded from percentage calculations unless otherwise indicated. q values were calculated using Benjamini–Hochberg FDR correction across variables tested in Table 2

Multivariable logistic regression analysis was performed to evaluate the associated factors of clinical severity (Table 3). Young age (age ≤ 9 years, OR 0.213, 95% CI 0.110–0.414) and (age = 10–25 years, OR 0.349, 95% CI 0.219–0.555), number of neurofibromas (1–9 neurofibromas, OR 0.157, 95% CI 0.083–0.298) and (10–99 neurofibromas, OR 0.299, 95% CI 0.156–0.573), and absence of learning difficulties (OR 0.483, 95% CI 0.277–0.842) were associated with mild phenotype. Presence of PN, bony abnormalities and learning disabilities is likely to be correlated with clinical severity. For example, fewer patients in DNB stage 1 carried PN (22.0% vs. 52.8%) and bony abnormalities (13.2% vs. 55.5%); more patients in DNB stage 5 carried PN (16.9% vs. 2.7%) and bony abnormalities (17.1% vs. 3.3%).

**Table 3:** Multivariable logistic regression analysis of factors associated with DNB-defined severe classification

Variables	β	Odds ratio	95%CI
**Gender**			
Male	-0.089	0.914	0.596 ~ 1.403
Female		REF	
**Age (years)**			
≤ 9	-1.547	0.213**	0.110 ~ 0.414
10–25	-1.053	0.349**	0.219 ~ 0.555
> 25		REF	
**Family history**			
Yes		REF	
No	-0.106	0.899	0.569 ~ 1.420
**Maximum diameter of CALMs (cm)**			
≤ 5		REF	
6 ~ 8	-0.021	0.980	0.574 ~ 1.671
> 8	-0.037	0.964	0.589 ~ 1.577
**Quantity of CALMs**			
0 ~ 9		REF	
10 ~ 99	0.645	1.907	0.986 ~ 3.689
> 99	0.215	1.239	0.617 ~ 2.489
**Quantity of neurofibromas**			
0		REF	
1 ~ 9	-1.850	0.157**	0.083 ~ 0.298
10 ~ 99	-1.206	0.299**	0.156 ~ 0.573
**Learning disabilities**			
Yes		REF	
No	-0.729	0.483*	0.277 ~ 0.842
**AN**			
Yes		REF	
No	0.329	1.390	0.780 ~ 2.475
**Halo-like phenomenon on dermal melanocytosis**			
Yes		REF	
No	0.271	1.311	0.769 ~ 2.236
**Age of onset**			
0	-0.183	0.833	0.182 ~ 3.803
> 0–2	0.416	1.515	0.400 ~ 5.737
≥ 2		REF	
**Age of onset of CALMs**			
0	0.404	1.498	0.294 ~ 7.638
1–4	-0.153	0.858	0.228 ~ 3.229
> 4		REF	
**Age of onset of neurofibromas**			
≤ 4	0.121	1.128	0.655 ~ 1.942
5–11	0.064	1.066	0.620 ~ 1.834
> 11		REF	

REF: reference group

OR < 1 indicates lower odds of DNB-defined severe classification

The category “>99 neurofibromas” was not estimated separately because of model instability

Given overlap between several clinical features and DNB staging, results are presented as associations with severity classification rather than independent prognostic predictors

*P < 0.05; **P < 0.01

A descriptive comparison of major clinical manifestations between the present cohort and selected published NF1 cohorts or reference estimates is provided in Supplement 4.

### Genotypic characteristics among patients undergoing genetic testing

**Fig. 1:**
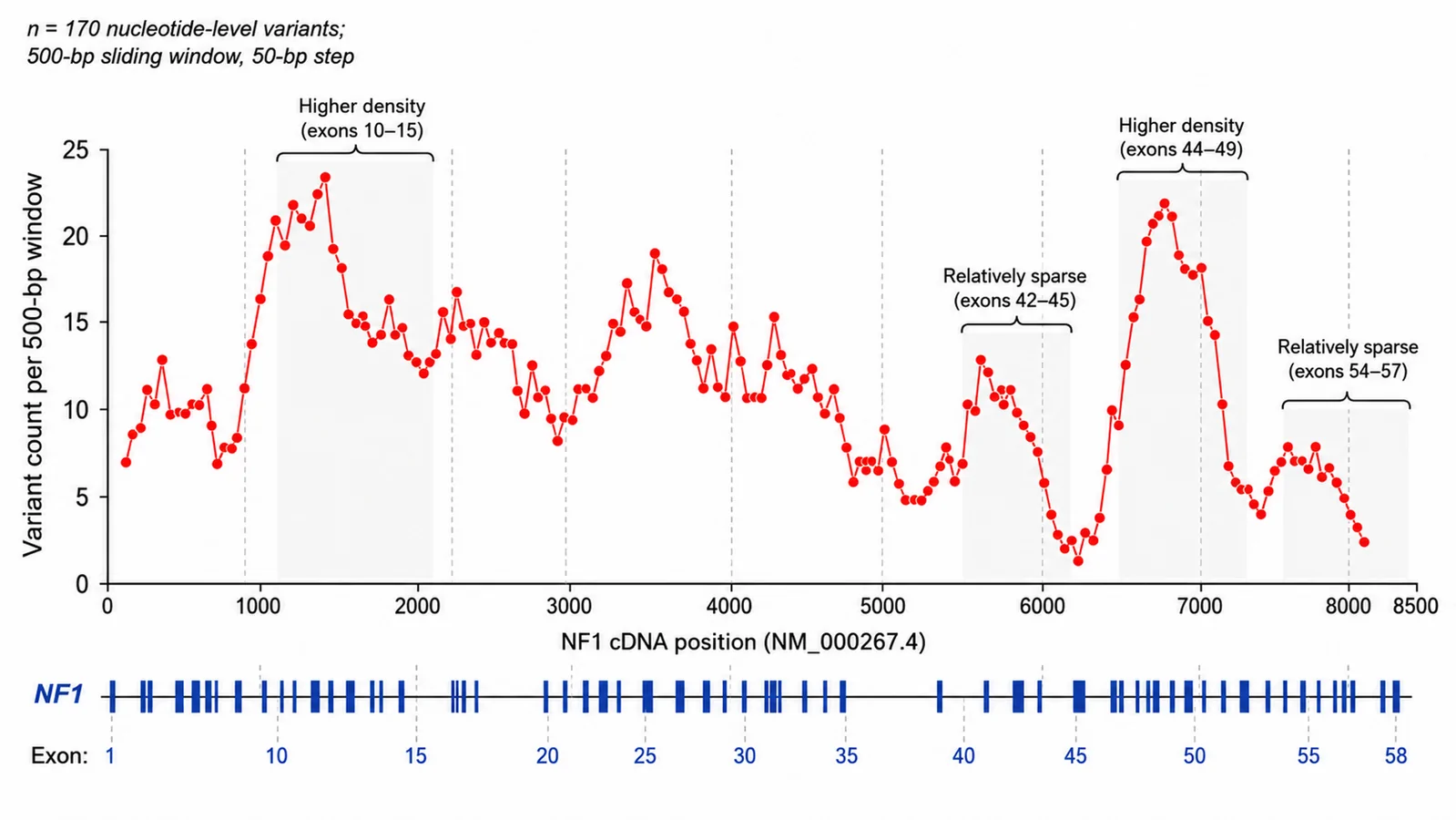
Transcript-level distribution of nucleotide-level *NF1* variants in the present cohort. Nucleotide-level NF1 variants were mapped to cDNA coordinates according to the reference transcript NM_000267.4. Exon-level deletions and duplications were excluded because their breakpoints could not be assigned to a single nucleotide-level position. Variant counts were calculated using a 500-bp sliding window with a 50-bp step across the NF1 cDNA sequence. The lower schematic shows the NF1 transcript structure, with blue vertical bars indicating exons. Regions with relatively higher variant counts were observed around exons 10–15 and exons 44–49, whereas relatively sparse regions were observed around exons 42–45 and exons 54–57. These patterns describe regional variation in reportable nucleotide-level NF1 variants rather than a recurrent hotspot or founder variant

Genetic testing was available for a subset of patients (*n* = 211). Among genetically characterized cases, a reportable *NF1* variant classified as pathogenic/likely pathogenic was identified in 182 (86.3%). Among the remaining 29 individuals without a reportable pathogenic/likely pathogenic *NF1* variant, 11 (5.2%) carried *NF1* variants of VUS, 18 (8.5%) tested negative for reportable variants. Genotype–phenotype observations were based on the 193 patients with detected *NF1* variants, including 182 with pathogenic/likely pathogenic variants and 11 with VUS. Among the 193 patients, 157 (81.3%) were sporadic cases and 36 (18.7%) had a family history of NF1. The identified variants included nonsense variants (30.6%), frameshift variants (23.8%), missense variants (18.1%), splice-site variants (14.0%), and exon-level/whole-gene deletions (10.9%), including one single-exon deletion, four multi-exon deletions, and 16 whole-gene deletions. Next-generation sequencing identified 152 distinct *NF1* variants in total, including 45 novel sites. A descriptive comparison of *NF1* genetic findings between the present cohort and selected published Asian, European, and global NF1 cohorts is provided in Supplement 5. After excluding exon-level deletions/duplications, 170 nucleotide-level variants were mapped to *NF1* cDNA coordinates. A 500-bp sliding-window density scan with a 50-bp step showed a non-uniform distribution of reportable nucleotide-level variants across the *NF1* transcript. The highest-density regions were located around exons 10–15, with a peak of approximately 23–24 variants per 500-bp window, and exons 44–49, with a peak of approximately 20–21 variants per 500-bp window. In contrast, relatively sparse regions were observed around exons 42–45 and near the transcript terminus around exons 54–57 (Fig. 1).

To contextualize these findings, we re-analyzed publicly available ClinVar *NF1* variants. After excluding intronic and UTR variants, 4,196 pathogenic/likely pathogenic variants, 2,398 benign/likely benign variants, and 5,349 variants of uncertain significance were retained. Pathogenic/likely pathogenic ClinVar variants showed a significantly non-uniform distribution across 16 non-overlapping 500-bp windows, with depletion in windows centered at cDNA positions 6251 and 7751 after FDR correction. By contrast, benign/likely benign variants did not show significant non-uniformity. When the window-level distribution of nucleotide-level variants in the present cohort was compared with ClinVar pathogenic/likely pathogenic variants, no differences remained significant after correction for multiple testing (Supplement 6). These findings suggest that the regional distribution observed in the present cohort broadly parallels the global distribution of pathogenic *NF1* variants rather than indicating an obvious population-specific founder effect.

The results of genetic testing and the corresponding clinical phenotypes are listed in Supplement 7. Of all 66 patients older than 20 years, 20 (30.3%) patients were classified as stage 1 (Table 4), and 4 of these 20 patients had no neurofibromas or other complications. In these four patients, three carried nonsense variants (c.3814 C > T, c.3826 C > T, and c.3520 C > T) distributed in exon 27 or 28, and one patient had an exon deletion. Of all 127 patients younger than 20 years, 11 (8.7%) patients were classified as stage 5. In eight of these 11 patients, variants were located in odd-numbered exons, including four variant sites in VD1 and four variant sites in VD5 (Table 4). Likewise, among all 193 patients, 13 patients with DNB stage 5 carried variants located in odd-numbered exons, including six variant sites in VD1 and four variant sites in VD5.

**Table 4:** Selected exploratory genotype–phenotype observations

Patient group	Variant type	Number of patients	Nucleotide and amino acid change
Older than 20 years and with DNB stage 1	nonsense	7 (6 distinct nucleotide changes)	c.1318 C > T p.(Arg440Ter), c.3814 C > T p.(Gln1272Ter), c.3826 C > T p.(Arg1276Ter), c.3520 C > T p.(Gln1174Ter), c.4265 C > G p.(Ser1422Ter), and c.5242 C > T p.(Arg1748Ter)
	exon deletions	4	exons 1–58
	missense	3	c.2540T > C p.(Leu847Pro), c.3827G > A p.(Arg1276Gln) and c.5546G > T p.(Arg1849Leu)
	splicing	3	c.1642–2 A > C, c.3871-1G > T, and c.6756 + 2T > C
	frameshift	2	c.3704delA p.(Gln1235ArgfsTer5) and c.7029delG p.(Asn2344IlefsTer31)
	indel	1	c.3639_3641del p.(Met1215del)
Younger than 20 years and with DNB stage 5	nonsense	5	c.1246 C > T p.(Arg416Ter), c.4108 C > T p.(Gln1370Ter), c.4357 C > T p.(Arg1513Ter), c.5839 C > T p.(Arg1947Ter), and c.6709 C > T p.(Arg2237Ter)
	frameshift	4	c.990_1041del p.(Ser331ProfsTer28), c.2126_2127delGC p.(Ala709GlnfsTer18), c.2731_2732insT p.(Leu911PhefsTer8), and c.5667dupT p.(Ile1890TyrfsTer2)
	splicing	2	c.5609 + 1G > A, c.4368-2G > A

To explore whether variant-related features were associated with DNB-defined severity among genetically characterized patients, we performed an exploratory multivariable analysis (Supplement 8). Clinical features overlapping with DNB staging showed the strongest associations. In contrast, VDs and predicted alterations in neurofibromin structure did not show robust independent associations with DNB-defined severity. Univariable analyses identified nominal associations between selected variant-related features and clinical parameters, but these did not constitute robust genotype–phenotype correlations after accounting for multiple testing, small subgroup sizes, and the cross-sectional design. (Supplement 9).

## Discussion

NF1 is a rare genetic disease with a significant degree of clinical heterogeneity caused by variants in the *NF1* gene. This cross-sectional study characterized the clinical features and *NF1* variant spectrum in a large Chinese cohort. We further contextualized the clinical and genetic findings of this Chinese cohort through comparison with previously reported cohorts and ClinVar-derived *NF1* variant data.

The present study has collected demographic and clinical information of 847 NF1 Chinese patients from 31 provinces, with an age range from 1 month to 70 years. Sporadic cases were accounted for 72.5% of all these NF1 patients, which is consistent with the *de novo* variant rate reported at 50%–85% in other studies [20, 35–39]. Consistent with a previously reported Japanese study, the age distribution of our NF1 cohort showed two peaks, with patients aged < 10 years and 20–40 years representing the most frequent age groups [31]. The fact that pediatric NF1 population (< 10 years) showed relatively high proportion of all patients was possibly due to the high desire of parents to seek medical care for their children as well as improved methods of clinical and genetic detection in recent years (screening and detection bias). Regarding the percentage of NF1 patients at different DNB stages, our study has shown similar results from the study by Yuko EHARA et al. for stage 1 (45.8% vs. 44.3%), stage 3 (1.65% vs. 2.4%), and stage 4 (1.2% vs. 0.8%) [31], but the percentage of patients at stage 5 in our study was lower (5.9% vs. 33.9%). In the correlation between age and disease severity, we observed that patients younger or older than 20 years are predominantly at stage 1 and stage 2, respectively. A higher proportion of DNB stage 5 classification was observed among patients aged 50–59 years. This may be due to historical relative lack of early detection of mild phenotypes as well as a population with lower desire to seek and access to medical care. It will be important to employ public education strategies to improve NF1-related literacy and offer welfare policies for complication screening; this will help identify and treat milder cases in older populations. Although the DNB classification provides a structured severity framework, it is not universally used outside Japan, and its components overlap with several clinical variables analyzed in this study. Future studies should incorporate internationally harmonized NF1 severity instruments, longitudinal follow-up, and standardized ophthalmologic, neurodevelopmental, and imaging assessments to improve clinical risk stratification.

Tadini et al. have summarized the proportion of clinical manifestations for NF1 patients worldwide [38]. In comparison, our study has observed similar proportion of manifestations including CALMs (98.3% vs. nearly 100%), neurofibromas (70.7% vs. 80%), PN (20.9% vs. 20%–50%), AN (20.2% vs. 20%–50%), and juvenile xanthogranulomas (JXGs, 16.3% vs. 10%–15%), and low proportion of manifestations including axillary and/or inguinal freckles (54.4% vs. 90%), learning disabilities (16.8% vs. 30%–60%), Lisch nodules (4.5% vs. 90% in adulthood and 50% in late childhood), optic pathway glioma (OPG, 7.3% vs. >10%), and other tumors. Our study has also indicated that the quantity of CALMs and cutaneous neurofibromas increase with age [39, 40]. 

To contextualize the clinical spectrum of the present Chinese NF1 cohort, we compared the frequencies of major clinical manifestations with selected published Asian, European, and reference NF1 datasets (Supplement 4). The frequencies of CALMs, neurofibromas, and PNs in the present cohort were broadly comparable with those reported in several East Asian and European cohorts. In contrast, Lisch nodules and OPG were reported less frequently in the present cohort than in several cohorts with more systematic ophthalmologic or imaging assessment. These differences may be partly attributable to variation in age structure, recruitment pathways, study design, and the completeness of ophthalmologic, neurodevelopmental, imaging, and complication screening.

Because severity in this cohort was operationalized using the DNB staging framework, two methodological features are important when evaluating the severity-stratified comparisons and multivariable models. First, NF1 is a progressive disorder; therefore, in a cross-sectional setting, the observed inverse association between younger age and severe classification is more likely to reflect differences in disease duration rather than an independent protective effect. Second, because severity was defined using the DNB framework, several clinical variables included in the regression model and severity-stratified comparisons (e.g., neurofibroma burden, learning difficulties, PN, and bony abnormalities) may overlap conceptually and/or operationally with components of the staging construct. Consequently, some differences observed between severity groups may reflect the construction of the DNB staging framework itself, limiting their use as independent prognostic predictors. Notably, PN was not uniformly associated with severe classification in our cohort, underscoring the marked phenotypic heterogeneity of NF1 and the multifactorial nature of severity assessment. Future studies with longitudinal follow-up, and analytical strategies that explicitly account for disease duration or incorporate sensitivity analyses excluding severity-defining components, will be important to more robustly delineate independent determinants of clinical progression. Inclusion of genetic diagnosis is one of the major changes in the NF1 diagnostic criteria in 2021 modified by the International Neurofibromatosis Diagnostic Criteria Consensus Group [41, 42]. Genetic counseling and prenatal/preimplantation diagnosis is strongly recommended for pregnant NF1 patients to reduce the likelihood of giving birth to an infant with NF1 [43, 44]. 

Several relatively large Chinese or Chinese-origin NF1 genetic cohorts have been reported. Lee et al. [45] analyzed 107 NF1 patients of Chinese origin and identified 45 novel and 23 known *NF1* variants. Zhang et al. [46] subsequently performed integrated genetic analysis in 109 mainland Chinese patients from 100 families with NF1-like phenotypes using Sanger sequencing, multiplex ligation-dependent probe amplification, and cDNA sequencing. More recently, Zhu et al. [47] reported a mainland Chinese cohort of 195 patients with NF1 who underwent genetic testing and systemic examination for genotype–phenotype correlation analysis. Compared with these previous studies, the present study includes a larger clinically ascertained cohort and provides additional data on the clinical and genetic spectrum of *NF1* in China.

To contextualize the genetic spectrum of this Chinese NF1 cohort, we compared our findings with selected Asian, European, and global NF1 cohorts reported in the literature (Supplement 5). Across studies, truncating variants, including nonsense and frameshift variants, constituted a major proportion of *NF1* variants, followed by missense and splice-site variants. The overall variant-class distribution in the present cohort was broadly consistent with previous NF1 genetic cohorts. Although 45 novel *NF1* variant sites were identified in our cohort, no recurrent founder variant or clearly population-specific mutational pattern was observed.

We further contextualized the transcript-level distribution of *NF1* variants using external ClinVar-derived data. Variant density was non-uniform across the *NF1* coding region; however, apparent regional clustering may reflect differences in variant ascertainment, sequencing strategy, copy-number variant detection, variant classification criteria, and ancestry annotation across datasets.

Although NF1 is generally considered to lack a single recurrent mutational hotspot, our transcript-level density analysis suggests that reportable nucleotide-level variants are not evenly distributed across the *NF1* cDNA sequence. Importantly, external comparison with ClinVar pathogenic/likely pathogenic variants showed no significant window-level difference after multiple-testing correction, suggesting that the distribution observed in our Chinese cohort is broadly consistent with the global pathogenic *NF1* variant landscape. Therefore, the observed density clustering is more consistent with regional enrichment of reportable nucleotide variants than with a Chinese- or Asian-specific hotspot or founder variant.

Several factors may contribute to apparent regional enrichment or depletion, including local sequence context, functional constraint, variant class, detection strategy, and variant classification practices. In ClinVar, pathogenic missense variants were enriched in the central GAP-related domain, whereas pathogenic frameshift variants, but not nonsense variants, showed depletion in the windows beginning at cDNA positions 6251 and 7751. These findings indicate that apparent regions of relatively higher or lower variant density may be variant-class dependent. The sparse region around exon 42 observed in our cohort is therefore intriguing and warrants further evaluation using analyses normalized for callable sequence length, sequencing coverage, and variant class, together with complementary copy-number variant, structural variant, and RNA-level analyses. Overall, these findings support comprehensive full-gene *NF1* testing rather than hotspot-based testing and underscore the need for multiethnic datasets generated using harmonized approaches for variant detection and classification.

A limited number of studies have reported correlations between *NF1* genotype and phenotype [48–52]. Patients with in-frame deletion variants in exon 17 (c.2970-2972delAAT and p.(Met922del)) were discovered to have a milder phenotype without neurofibromas, and missense variants affecting the Arg1809 associated with learning disabilities, pulmonic stenosis, and high potential of developing Noonan syndrome features, but without PN [53–56]. Such variants were undetectable in our patient population. Another study has reported that patients with severe phenotype associated with missense variants at codons 844–848 could present with superficial PN, symptomatic spinal cord neurofibroma, OPG, bony abnormalities, and a high risk of malignancy [57]. Zhu et al. found that patients with splicing variants exhibited a significantly higher prevalence of spinal PN than those with truncating variants [47]. In our study, a female patient aged one year with the p.(Leu844Phe) variant was born with scoliosis. However, both a 20-year-old male patient and a one-year-old female patient carrying the *NF1* p.(Leu847Pro) variant presented only abnormal skin pigmentation. These patients will be monitored over time as well as screened regularly for complications. Patients with *NF1* exon deletions are frequently related to more severe phenotypes including massive neurofibromas, intellectual disability, cardiovascular malformations, and increased risk of malignant peripheral nerve sheath tumors [58]. This variant class was identified in 21 patients in the present cohort. This study has also identified several variants (Table 4) carried by younger patients with higher DNB stage. Although missense variants are generally considered less disruptive to neurofibromin than truncating variants, a 10-year-old patient with the c.6881T > G missense variant in our cohort had a severe clinical presentation, including thousands of neurofibromas, PN, scoliosis, and learning disabilities. This highlights that although genotype–phenotype correlations may exist between alterations in neurofibromin and symptom onset, much remains to be understood about the pathobiology of *NF1* and the determinants of clinical severity.

While this study provides new information about NF1, there are limitations. First, this was not a population-based study. A substantial proportion of participants were recruited through a national patient advocacy organization, which may have enriched the cohort for patients and families with greater disease awareness, stronger healthcare-seeking behavior, or more prominent clinical manifestations. Therefore, the observed frequencies of clinical features and the age distribution are best regarded as characteristics of a clinically ascertained cohort rather than population prevalence estimates for NF1 in China. Nevertheless, this recruitment strategy enabled enrollment of patients from a broad geographic range and provided a valuable real-world description of clinically ascertained Chinese patients with NF1. Second, despite the relatively large sample size, some patients did not complete clinical testing such as eye examinations, complication screening, and thus, clinical information for these patients was incomplete. Third, genetic testing was performed only in a subset of patients, limiting the statistical power of genotype–phenotype analyses. Fourth, WES may not fully detect deep intronic variants, low-level mosaicism, complex structural variants, or RNA-level splicing effects. Future studies with larger genetically characterized cohorts, longitudinal follow-up, and comprehensive variant detection will be needed to validate clinically meaningful genotype–phenotype relationships in NF1.

## Conclusion

This cross-sectional study summarizes the clinical and genetic features of Chinese patients with NF1 and expands the known *NF1* variant spectrum in this population. Transcript-level analysis showed regional variation in reportable *NF1* variants, which broadly paralleled the ClinVar pathogenic/likely pathogenic *NF1* variant distribution. These findings provide a basis for future longitudinal studies to validate clinically meaningful genotype–phenotype relationships in NF1.

## Supplementary Information

Below is the link to the electronic supplementary material.

Supplementary Material 1

Supplementary Material 2

Supplementary Material 3

Supplementary Material 4

Supplementary Material 5

Supplementary Material 6

Supplementary Material 7

Supplementary Material 8

Supplementary Material 9

## Data Availability

All data generated or analyzed during this study are included in this published article and its supplementary information files.

## Abbreviations

NF1: Neurofibromatosis type 1
CALMs: Café-au-lait macules
HGMD: The Human Gene Mutation Database
WES: Whole-exome sequencing
VUS: Variant of uncertain significance
UTR: Untranslated region
VD: Variant domain
OR: Odds ratio
CI: Confidence interval
FDR: False-discovery rate
PN: Plexiform neurofibroma
AN: Nevus anemicus
JXG: Juvenile xanthogranuloma
OPG: Optic pathway glioma
