# Supplementary material for: Clinical characteristics and variant spectrum of NF1 in Chinese patients with neurofibromatosis type 1: a cross-sectional study
Source: Orphanet J Rare Dis. 2026 Aug 17;21:416. doi: 10.1186/s13023-026-04534-9 (PMC13756884; doi:10.1186/s13023-026-04534-9)
Supplement: Supplementary file 1 [file 13023_2026_4534_MOESM1_ESM.docx]

**Supplement 1. Clinical questionnaire used for data collection**

Patient Number：

**Demographic Information**

1. Patient origin: ① 1= Outpatient clinics ② 2= The Neurofibromatosis Care Center

2. Gender: ① 1= Male ② 2= Female
3. Age: (years)
4. Ethnicity: ① 1= Han ② 2= Non-Han minorities
5. Place of birth:
6. Occupation:
7. E-mail:
8. Age of onset:  (years)
9. Disease course: (years)
10. Family history: ① 1= Yes ② 2= No
11. Treatment: ① 1= Yes and specific treatment options: ② 2= No

**Café-au-lait macules characteristics**

1. Café-au-lait macules: ① 1= Yes ② 2= No
2. Age of appearance of café-au-lait macules:
3. Areas of café-au-lait macules involved in the onset of the disease: ① Head, face and neck  ② Trunk ③ Upper Extremities ④ Lower Extremities ⑤ The whole body
4. Age at which café-au-lait macules increase significantly:  (years)
5. Maximum diameter of the current café-au-lait macules: (cm)
6. Approximate number of café-au-lait macules at present:
7. Areas of café-au-lait macule currently involved: ① Head, face and neck ② Trunk ③ Upper Extremities ④ Lower Extremities ⑤ The whole body

**Neurofibromas characteristics**

1. Neurofibromas: ① 1= Yes ② 2= No
2. Age of appearance of neurofibromas:
3. Areas of neurofibromas involved in the onset of the disease: ① Head, face and neck ② Trunk ③ Upper Extremities ④ Lower Extremities ⑤ The whole body
4. Age at which neurofibromas increase significantly: (years)
5. Approximate number of neurofibromas at present:
6. Areas of neurofibromas currently involved: ① Head, face and neck ② Trunk  ③ Upper Extremities ④ Lower Extremities ⑤ The whole body
7. Plexiform neurofibromas: ① 1= Yes ② 2= No
8. Abnormal sensation of neurofibromas: ① 1= Yes ② 2= No

**Other manifestations**

1. Axillary and/or inguinal freckles: ① 1= Yes ② 2= No
2. Bony abnormalities: ① 1= Yes: ② 2= No
3. Lisch nodules: ① 1= Yes ② 2= No ③ 3= not checked
4. Learning disabilities: ① 1= Yes ② 2= No
5. Nevus anemicus: ① 1= Yes ② 2= No
6. Juvenile xanthogranulomas: ① 1= Yes ② 2= No
7. Halo-like phenomenon on dermal melanocytosis: ① 1= Yes ② 2= No
8. Optic pathway glioma, epilepsy, meningioma, schwannoma, and other spinal cord tumors: ① 1= Yes ② 2= No ③ 3= not checked

**Others**

Photographs:

Genetic testing: ① 1= Yes and results: ② 2= No

Any additional information:
