# Supplementary material for: Clinical characteristics and variant spectrum of NF1 in Chinese patients with neurofibromatosis type 1: a cross-sectional study
Source: Orphanet J Rare Dis. 2026 Aug 17;21:416. doi: 10.1186/s13023-026-04534-9 (PMC13756884; doi:10.1186/s13023-026-04534-9)
Supplement: Supplementary file 2 [file 13023_2026_4534_MOESM2_ESM.docx]

**Supplement 2. Grade of severity of neurofibromatosis 1 by Japanese DNB classification**

| Stage | DNB classification | | | Life function and social activity |
| --- | --- | --- | --- | --- |
|  | D | N | B |  |
| 1 | D1 | N0 | B0 | No problems in daily life and social activity |
| 2 | D1-2 | N0 | B1 | Mild problems in daily life and social activity |
|  |  | N1 | B0-1 |  |
| 3 | D3 | N0 | B0 | Mild problems in daily life and severe problems in social activity |
| 4 | D3 | N1 | B0-1 | Moderate problems in daily life and severe problems in social activity |
|  | D3 | N0-1 | B1 |  |
| 5 | D4 | AnyN | AnyB | Severe problems in daily life due to physical abnormality |
|  | AnyD | N2 | AnyB |  |
|  | AnyD | AnyN | B2 |  |

Dermatological manifestations

| D1 | Pigmented macules and a few neurofibromas |
| --- | --- |
| D2 | Pigmented macules and many neurofibromas |
| D3 | Numerous neurofibromas (>1000 in number, >1 cm in size) |
| D4 | Severe plexiform neurofibromas or malignant peripheral nerve sheath tumor |

Neurological manifestations

| N0 | No neurological symptoms |
| --- | --- |
| N1 | Neurological symptoms (e.g. paralysis or pain) or/and abnormal neurological findings |
| N2 | Severe or progressive neurological symptoms |

Bone manifestations

| B0 | No bone lesion |
| --- | --- |
| B1 | Mild or moderate bone lesion (deformity in spine or extremities that does not need treatment) |
| B2 | Severe bone lesion (dystrophic type or spine deformity that needs surgical treatment [e.g. scoliosis or kyphosis]), severe bone deformity in extremities (e.g. pseudarthrosis, fracture) or defect of the skull or facial bone |
