# Supplementary material for: Clinical characteristics and variant spectrum of NF1 in Chinese patients with neurofibromatosis type 1: a cross-sectional study
Source: Orphanet J Rare Dis. 2026 Aug 17;21:416. doi: 10.1186/s13023-026-04534-9 (PMC13756884; doi:10.1186/s13023-026-04534-9)
Supplement: Supplementary file 3 [file 13023_2026_4534_MOESM3_ESM.docx]

**Supplement 3. Age and sex distribution of 847 patients with NF1**

| **Age group** | **Male (%)** | **Female (%)** | **Total** |
| --- | --- | --- | --- |
| 0~9 | 154 (51.0) | 148 (49.0) | 302 |
| 10~19 | 43 (45.7) | 51 (54.3) | 94 |
| 20~29 | 70 (31.8) | 150 (68.2) | 220 |
| 30~39 | 66 (39.8) | 100 (60.2) | 166 |
| 40~49 | 18 (42.9) | 24 (57.1) | 42 |
| 50~59 | 7 (38.9) | 11 (61.1) | 18 |
| ≥60 | 1 (20.0) | 4 (80.0) | 5 |
| Total | 359 (42.4) | 488 (57.6) | 847 |
