# Supplementary material for: Clinical characteristics and variant spectrum of NF1 in Chinese patients with neurofibromatosis type 1: a cross-sectional study
Source: Orphanet J Rare Dis. 2026 Aug 17;21:416. doi: 10.1186/s13023-026-04534-9 (PMC13756884; doi:10.1186/s13023-026-04534-9)
Supplement: Supplementary file 4 [file 13023_2026_4534_MOESM4_ESM.docx]

**Table S4 Comparison of major clinical manifestations between the present Chinese NF1 cohort and selected published NF1 cohorts or reference estimates**

| Study | Region &  Study type | N | Age | CALMs | axillary and/or inguinal freckles | Neurofibromas | PN | Lisch nodules | learning disabilities/neurodevelopmental manifestations | Skeletal abnormalities | OPG |
| --- | --- | --- | --- | --- | --- | --- | --- | --- | --- | --- | --- |
| Present study | China & Cross-sectional cohort | 847 | Median age of 23 (range: 1 month–70 years) | 98.3% | 54.4% | 70.7% | 20.9% | 4.5% | 16.8% | 17.9% | 7.3% |
| Kim& Cheon,2014^1^ | Korea & Single-center case series | 42 | 15.8±14.6 (range: 1–62 years) | 100% | 52.4% | 73.8% | 9.5% | 16.7% | Mental retardation (11.9%);  Seizures (2.4%) | Scoliosis (28.6%);  Pseudoarthrosis  (2.4%) | 4.8% |
| Bashiri et al., 2020^2^ | Saudi Arabia & observational retrospective cohort | 50 | Mean age of 11 (range: 1-18 years) | 90% | 34% | 12% | 24% | 42% | Epilepsy (21.2%);  Intellectual disability (18.1%);  Learning disabilities (6.1%) | Scoliosis (27.3%);  Pseudarthrosis (3%) | 34% |

**Table S4 Comparison of major clinical manifestations between the present Chinese NF1 cohort and selected published NF1 cohorts or reference estimates(continued)**

| Study | Region &  Study type | N | Age | CALMs | axillary and/or inguinal freckles | Neuro fibromas | PN | Lisch nodules | learning disabilities/neurodevelopmental manifestations | Skeletal abnormalities | OPG |
| --- | --- | --- | --- | --- | --- | --- | --- | --- | --- | --- | --- |
| Scala et al., 2021^3^ | Different ancestries & Single-center cohort | 365 fulfilled NIH criteria | 18.5±12.86 (range:1–71years) | 96.2% | 76.4% | 36.7% | 20.5% | 23.8% | 48.5% | 35.9% | 23.8% |
| GeneReviews,Friedman^4^ | Expert-curated reference summary | NR | Feature frequencies vary strongly by age | >99% | 85% | cNFs 99% in adolescence/adulthood;  nodular neurofibroma (~15%) | ~30% | >95% | Learning difficulties (50%-60%)  Intellectual disability (4%-8%)  Seizures  (6%-7%) | Long bone dysplasia  (2%)  dystrophic scoliosis (5%); nondystrophic scoliosis (5%) | 15%-20% |

**Abbreviations:** CALMs: café-au-lait macules; PN: plexiform neurofibromas; OPG: optic pathway glioma; cNFs: cutaneous neurofibromas; NR: not reported or not extractable from the reported data.

Values were extracted from published cohorts or reference summaries and are not directly comparable because of differences in age structure, recruitment strategy, diagnostic criteria, study design, and completeness of ophthalmologic, neurodevelopmental, imaging, and complication screening. Percentages were calculated using the denominators reported in the original studies. NR indicates not reported or not extractable from the published data.

**Reference:**

1.	Kim MJ, Cheon CK. Neurofibromatosis type 1: a single center's experience in Korea. *Korean J Pediatr* 2014; **57**(9): 410-5.

2.	Bashiri FA, AlZamil LR, Aldhuwayhi RA. Clinical spectrum of neurofibromatosis type 1 among children in a tertiary care center. *Neurosciences (Riyadh)* 2020; **25**(5): 375-9.

3.	Scala M, Schiavetti I, Madia F, et al. Genotype-Phenotype Correlations in Neurofibromatosis Type 1: A Single-Center Cohort Study. *Cancers (Basel)* 2021; **13**(8).

4.	Friedman JM. Neurofibromatosis 1. In: Adam MP, Feldman J, Mirzaa GMea, eds. GeneReviews®. Seattle (WA): University of Washington, Seattle; 2025.
