# Supplementary material for: Clinical characteristics and variant spectrum of NF1 in Chinese patients with neurofibromatosis type 1: a cross-sectional study
Source: Orphanet J Rare Dis. 2026 Aug 17;21:416. doi: 10.1186/s13023-026-04534-9 (PMC13756884; doi:10.1186/s13023-026-04534-9)
Supplement: Supplementary file 5 [file 13023_2026_4534_MOESM5_ESM.docx]

**Table S5 Comparison of NF1 genetic findings between the present Chinese cohort and selected published Asian, European, and global NF1 cohorts**

| Study | Region/  population | Cohort size | Genetic testing approach | NF1 detection rate/ Reported variants | Main variant classes or key findings | Novel variants | Hotspot /founder pattern |
| --- | --- | --- | --- | --- | --- | --- | --- |
| Present study | China | 211 patients included in the *NF1*-specific genetic summary; 193 patients with detected *NF1* variants | Whole-exome sequencing; variant classification according to ACMG/AMP guidelines; Sanger validation in a subset | Pathogenic/likely pathogenic *NF1* variants were identified in 182/211 patients (86.3%); 11/211 patients (5.2%) carried *NF1* variants of uncertain significance; 18/211 patients (8.5%) had no reportable *NF1* variant | Nonsense 30.6%,  frameshift 23.8%,  missense 18.1%,  splice-site 14.0%,  exon deletions 10.9%;  152 distinct *NF1* variants; non-uniform transcript-level distribution with clustering in exons 10–15 and 44–49 | 45 novel sites | No recurrent founder variant was identified; the observed clustering is more consistent with regional enrichment of reportable nucleotide-level variants than with a definitive population-specific hotspot |
| Lee et al., 2006^1^ | Taiwanese/Chinese NF1 patients | 107 patients | NF1 mutation screening | Identified 45 novel and 23 known NF1 mutations | Nonsense (20),  frameshift (23),  splice-site (9),  missense (11),  gross deletion variants (5);  Most pathogenic events were truncating mutations predicted to generate premature termination codons | 45 novel sites | No definitive founder mutation established |

**Table S5 Comparison of NF1 genetic findings between the present Chinese cohort and selected published Asian, European, and global NF1 cohorts**

**(continued)**

| Study | Region/  population | Cohort size | Genetic testing approach | NF1 detection rate/ Reported variants | Main variant classes or key findings | Novel variants | Hotspot /founder pattern |
| --- | --- | --- | --- | --- | --- | --- | --- |
| Zhang et al., 2015^2^ | Mainland China | 109 patients from 100 families with NF1-like phenotypes | Sanger sequencing, MLPA, and cDNA sequencing | NF1 mutations identified in 97 individuals; detection rate 89% in NF1-like probands and 93% among subjects fulfilling NIH criteria | Nonsense (21), frameshift (26),  splicing (15),  Missense (12),  in-frame deletions (2),  indel (1),  microdeletions (11), and single-exon deletion (1);  exon 15 showed high size-adjusted mutation frequency | 34 novel intragenic mutations | No obvious mutation hotspot; exon 15 enrichment noted after exon-size weighting |
| Ko et al., 2013^3^ | Korea | 78 patients from 60 families | Entire coding region and flanking splice-site analysis, combined with RT-PCR, MLPA, and/or FISH | 52 distinct NF1 mutations identified in 60 families | Missense mutations 23.1%,  Nonsense mutations 34.6%,  Splice-site mutations 21.2%,  Small deletions 7.7%,  Small insertions 5.8%,  Gross deletions / large deletions 7.7% | 16 novel mutations | Lack of a mutational hot spot; some common alleles suggesting founder effects |

**Table S5 Comparison of NF1 genetic findings between the present Chinese cohort and selected published Asian, European, and global NF1 cohorts**

**(continued)**

| Study | Region/  population | Cohort size | Genetic testing approach | NF1 detection rate/ Reported variants | Main variant classes or key findings | Novel variants | Hotspot /founder pattern |
| --- | --- | --- | --- | --- | --- | --- | --- |
| Kang et al., 2020^4^ | Korea | 427 Korean NF1 patients from 389 unrelated families | Long-range PCR; Sanger sequencing; CNV/whole-gene deletion | NF1 mutations in 339 families (87.1%) | Frameshift 33.6%,  Nonsense 28.9%,  Missense 15.3%,  Splicing 11.2%  Large deletion 7.1%,  In-frame indel  3.8% | 94(37.6%) mutations | No obvious mutation hotspot; 38 recurrent mutations |
| Futagawa et al., 2024^5^ | Japan | 44 suspected NF1 patients | NGS panel and CNV analysis | Germline pathogenic variants detected in 36/44 (81.8%); detection rate 92.3% among patients meeting revised NIH criteria | Nonsense 44.5%,  Frameshift 22.2%,  Splice 19.4%,  Large deletions 8.3%,  Missense 5.6% | 20.7% of variants novel | No population founder pattern reported |
| Scala et al., 2021^6^ | European single-center cohort | 583 individuals with ≥1 NIH criterion;  365 fulfilled ≥2 NIH criteria | cDNA-based sequencing, NGS, and MLPA | Detection rate 78.6%,  SNV 73.2%  CNV 5.5%  pediatric subgroup detection rate = 80.4% | SNV: Stop-gain 30.7%,  Missense 26.1%,  Frameshift 23.6%,  Splicing 15.0%,  CNV: Whole-gene deletion 48%,  Partial deletion 44%, Duplication 8% | NR | Recurrent variants existed, but no identified hotspot/ founder pattern |

**Table S5 Comparison of NF1 genetic findings between the present Chinese cohort and selected published Asian, European, and global NF1 cohorts**

**(continued)**

| Study | Region/  population | Cohort size | Genetic testing approach | NF1 detection rate/ Reported variants | Main variant classes or key findings | Novel variants | Hotspot /founder pattern |
| --- | --- | --- | --- | --- | --- | --- | --- |
| Sabbagh et al., 2013 ^7^— NF-France Network | France | 565 unrelated patients | RT-PCR, cDNA sequencing, and MLPA | Detection rate ≈ 97% | Frameshift 29%,  Splice frameshift 19.6%,  Nonsense 20.9%,  Missense 7.5%,  Splice in-frame 6.5%,  Single/multi-exon del/dup 3.9%,  Whole NF1 deletion 4.2%,  Complex mutations 3.9% | 363 distinct point mutations | No hotspot domain and founder pattern identified |
| van Minkelen et al., 2014^8^ | Netherlands | 1,985 index patients undergoing molecular diagnostics | genomic DNA–based approach | Overall detection rate = 59.3%; NIH criteria-positive subgroup: detection rate = 80.9% | Frameshift 31.7%  Nonsense 29.4%,  Splicing 19.7%,  Missense 9.8%,  NF1 microdeletions 5.3%,  Other 4.1% | unclassified variants 7.3% & LOVD database | Without clear hotspots |
| Mattocks et al., 2004^9^ | European diagnostic cohort | 167 subjects;  91 fulfilled ≥2 NIH criteria | PCR, Sanger sequencing, and ACSA | Detection rate 89.0% | Single nucleotide substitutions (61),  Frameshift (21),  Splice-site (10),  In-frame deletion (4) | 74% novel | Exons 11–17 cluster |

**Abbreviations:** NF1, neurofibromatosis type 1; P/LP, pathogenic/likely pathogenic; VUS, variant of uncertain significance; MLPA, multiplex ligation-dependent probe amplification; CNV, copy-number variant; WES, whole-exome sequencing; NGS, next-generation sequencing; RT-PCR, reverse-transcription polymerase chain reaction, ACSA, Automated Comparative Sequence Analysis; NR: not reported or not extractable from the reported data; ACMG/AMP, American College of Medical Genetics and Genomics/Association for Molecular Pathology.

Genetic testing approaches, inclusion criteria, variant classes, and reporting practices differed across studies; therefore, detection rates and variant-class distributions should be compared descriptively. For the present study, 211 patients were included in the NF1-specific genetic summary, and 193 patients had detected NF1 variants, including 182 with pathogenic/likely pathogenic variants and 11 with variants of uncertain significance.

**Reference:**

1.	Lee MJ, Su YN, You HL, et al. Identification of forty-five novel and twenty-three known NF1 mutations in Chinese patients with neurofibromatosis type 1. *Hum Mutat* 2006; **27**(8): 832.

2.	Zhang J, Tong H, Fu X, et al. Molecular Characterization of NF1 and Neurofibromatosis Type 1 Genotype-Phenotype Correlations in a Chinese Population. *Sci Rep* 2015; **5**: 11291.

3.	Ko JM, Sohn YB, Jeong SY, Kim HJ, Messiaen LM. Mutation spectrum of NF1 and clinical characteristics in 78 Korean patients with neurofibromatosis type 1. *Pediatr Neurol* 2013; **48**(6): 447-53.

4.	Kang E, Kim YM, Seo GH, et al. Phenotype categorization of neurofibromatosis type I and correlation to NF1 mutation types. *J Hum Genet* 2020; **65**(2): 79-89.

5.	Futagawa M, Okazaki T, Nakata E, et al. Genotypes and phenotypes of neurofibromatosis type 1 patients in Japan: A Hereditary Tumor Cohort Study. *Hum Genome Var* 2024; **11**(1): 42.

6.	Scala M, Schiavetti I, Madia F, et al. Genotype-Phenotype Correlations in Neurofibromatosis Type 1: A Single-Center Cohort Study. *Cancers (Basel)* 2021; **13**(8).

7.	Sabbagh A, Pasmant E, Imbard A, et al. NF1 molecular characterization and neurofibromatosis type I genotype-phenotype correlation: the French experience. *Hum Mutat* 2013; **34**(11): 1510-8.

8.	van Minkelen R, van Bever Y, Kromosoeto JN, et al. A clinical and genetic overview of 18 years neurofibromatosis type 1 molecular diagnostics in the Netherlands. *Clin Genet* 2014; **85**(4): 318-27.

9.	Mattocks C, Baralle D, Tarpey P, ffrench-Constant C, Bobrow M, Whittaker J. Automated comparative sequence analysis identifies mutations in 89% of NF1 patients and confirms a mutation cluster in exons 11-17 distinct from the GAP related domain. *J Med Genet* 2004; **41**(4): e48.
