# Supplementary material for: Clinical characteristics and variant spectrum of NF1 in Chinese patients with neurofibromatosis type 1: a cross-sectional study
Source: Orphanet J Rare Dis. 2026 Aug 17;21:416. doi: 10.1186/s13023-026-04534-9 (PMC13756884; doi:10.1186/s13023-026-04534-9)
Supplement: Supplementary file 6 [file 13023_2026_4534_MOESM7_ESM.docx]

**Supplement 7. Genotypic characteristics and associated clinical features of 193 patients with detected NF1 variants**

| Patient No. | Gender | Age | Nucleotide change | Amino acid change | Variant type | Pathogenicity | Location | Novelty | Family history | Note | Clinical characteristics | | | |
| --- | --- | --- | --- | --- | --- | --- | --- | --- | --- | --- | --- | --- | --- | --- |
|  |  |  |  |  |  |  |  |  |  |  | Severity | CALMs | Neurofibromas | Other manifestations |
| 1 | F | 7 | c.3118A>T | p.(Lys1040Ter) | Nonsense | P | E24 | Novel | － |  | 1 | II | － | Axillary and/or inguinal freckles, Learning disabilities |
| 2 | M | 9 | c.4357C>T | p.(Arg1513Ter) | Nonsense | P | E35 | Novel | － |  | 5 | II | － | Bony abnormalities |
| 3 | F | 23 | c.2728G>T | p.(Gly910Ter) | Nonsense | P | E21 | Novel | － |  | 2 | III | II | Axillary and/or inguinal freckles, Lisch nodules, Bony abnormalities |
| 4 | M | 1 | c.4259T>G | p.(Leu1420Ter) | Nonsense | P | E31 | Novel | － |  | 1 | II | － | AN |
| 5 | F | 24 | c.1845G>C | p.(Lys615Asn) | Missense | P | E16 | Novel | － |  | 2 | II | I | Lisch nodules, Tibial curvature, Scoliosis, AN |
| 6 | F | 1 | c.4862T>G | p.(Val1621Gly) | Missense | LP | E36 | Novel | ＋ |  | 1 | I | － | － |
| 7 | F | 11 | c.2126_2127delGC | p.(Ala709GlnfsTer18) | Frameshift | P | E18 | Novel | － |  | 5 | II | III | PN, Axillary and/or inguinal freckles |
| 8 | F | 6 | c.3376_3377delCA | p.(Gln1126AsnfsTer68) | Frameshift | LP | E26 | Novel | － |  | 1 | II | － | Lisch nodules, Halo-like phenomenon on Dermal melanocytosis, JXGs |
| 9 | F | 24 | c.2654_2655insAAACGCAGATACACCTGTCA | p.(Gly885GlyfsTer24) | Frameshift | LP | E21 | Novel | ＋ |  | 5 | II | I | PN, Axillary and/or inguinal freckles, Scoliosis |
| 10 | F | 29 | c.2654_2655insAAACGCAGATACACCTGTCA | p.(Gly885GlyfsTer24) | Frameshift | LP | E21 | Novel | － |  | 5 | III | II | PN, Axillary and/or inguinal freckles, Halo-like phenomenon on Dermal melanocytosis, JXGs |
| 11 | F | 8 | c.2731_2732insT | p.(Leu911LeufsTer8) | Frameshift | LP | E21 | Novel | － |  | 5 | II | I | PN, Axillary and/or inguinal freckles, Lisch nodules, Funnel chest, Scoliosis, Tibial curvature, AN, Halo-like phenomenon on Dermal melanocytosis |
| 12 | F | 32 | c.2875_2876insA | p.(Gln959GlnfsTer16) | Frameshift | P | E22 | Novel | ＋ |  | 5 | II | I | PN, Axillary and/or inguinal freckles |
| 13 | F | 23 | c.3704delA | p.(Gln1235ArgfsTer5) | Frameshift | LP | E27 | Novel | － |  | 1 | II | II | Axillary and/or inguinal freckles |
| 14 | F | 3 | c.403_404delCGinsA | p.(Arg135ArgfsTer30) | Frameshift | P | E4 | Novel | ＋ |  | 2 | II | II | Eye obliquity and nystagmus, Learning disabilities, AN |
| 15 | M | 1 | c.4070_4071delTCinsA | p.(Phe1357TyrfsTer28) | Frameshift | P | E30 | Novel | － |  | 2 | II | － | Tibial pseudarthrosis |
| 16 | F | 27 | c.5822delA | p.(His1941LeufsTer17) | Frameshift | LP | E40 | Novel | － |  | 2 | II | I | － |
| 17 | F | 31 | c.2606_2609delCAGT | p.(Ser871AsnfsTer6) | Frameshift | LP | E21 | Novel | － |  | 4 | II | III | Lisch nodules, Learning disabilities |
| 18 | M | 25 | c.1642_1643insG | p.(Ala548GlyfsTer10) | Frameshift | P | E15 | Novel | － |  | 2 | II | II | － |
| 19 | M | 38 | c.1950dup | p.(Leu651ThrfsTer19) | Frameshift | P | E17 | Novel | － |  | 2 | － | II | － |
| 20 | M | 14 | c.1959delT | p.(Pro654LeufsTer34) | Frameshift | P | E17 | Novel | － |  | 1 | II | II | Axillary and/or inguinal freckles |
| 21 | F | 29 | c.2199delC | p.(Asn734ThrfsTer14) | Frameshift | P | E18 | Novel | － |  | 4 | III | IV | Spinal cord tumors, AN |
| 22 | F | 7 | c.2237delA | p.(Asn746IlefsTer2) | Frameshift | P | E18 | Novel | － |  | 1 | II | － | Axillary and/or inguinal freckles |
| 23 | F | 7 | c.237delAinsTT | p.(Leu79PhefsTer28) | Frameshift | P | E3 | Novel | － |  | 1 | II | I | JXGs |
| 24 | F | 1 | c.3079_3083dup | p.(Asp1028GlufsTer10) | Frameshift | P | E23 | Novel | － |  | 1 | II | － | － |
| 25 | M | 3 | c.3245delG | p.(Gly1082fs) | Frameshift | LP | E25 | Novel | － |  | 1 | II | － | AN, JXGs |
| 26 | F | 9 | c.3250_3254dup | p.(Gln1086LeufsTer12) | Frameshift | P | E25 | Novel | － |  | 2 | II | I | Axillary and/or inguinal freckles, Learning disabilities, Halo-like phenomenon on Dermal melanocytosis |
| 27 | M | 32 | c.7029delG | p.(Asn2344IlefsTer31) | Frameshift | P | E47 | Novel | － |  | 1 | I | II | Axillary and/or inguinal freckles |
| 28 | M | 15 | c.7054dup | p.(Gln2352ProfsTer13) | Frameshift | LP | E47 | Novel | ＋ | paternal | 2 | II | II | － |
| 29 | M | 5 | c.7768dupA | p.(Thr2590AsnfsTer2) | Frameshift | P | E53 | Novel | － |  | 2 | II | － | Axillary and/or inguinal freckles, Developmental delay |
| 30 | F | 14 | c.990_1041del | p.(Ser331ProfsTer28) | Frameshift | P | E9 | Novel | － |  | 5 | II | I | PN |
| 31 | M | 27 | c.1576_1579dup | p.(Thr527AsnfsTer32) | Frameshift | LP | E14 | Novel | － |  | 2 | II | II | － |
| 32 | F | 33 | c.2208delinsTA | p.(Thr737AsnfsTer31) | Frameshift | LP | E18 | Novel | － |  | 5 | II | II | PN, Axillary and/or inguinal freckles, Arm flexion, Halo-like phenomenon on Dermal melanocytosis |
| 33 | M | 36 | c.5852delC | p.(Thr1951MetfsTer7) | Frameshift | LP | E40 | Novel | － |  | 2 | II | － | Axillary and/or inguinal freckles, Tibial pseudarthrosis, AN |
| 34 | F | 2 | c.5911_5912del | p.(Ile1971SerfsTer16) | Frameshift | P | E40 | Novel | － |  | 1 | III | － | Axillary and/or inguinal freckles, AN, Halo-like phenomenon on Dermal melanocytosis |
| 35 | F | 11 | del | － | Exon deletion | P | E48~49 | Novel | － |  | 2 | II | － | Axillary and/or inguinal freckles, Scoliosis |
| 36 | M | 18 | c.5609+1G>A | － | Splicing | P | I38 | Novel | － |  | 5 | II | I | PN |
| 37 | F | 31 | c.1642-2A>C | － | Splicing | LP | I14 | Novel | － |  | 1 | II | I | Axillary and/or inguinal freckles, AN |
| 38 | F | 2 | c.730+1G>T | － | Splicing | P | I7 | Novel | － |  | 2 | II | I | Scoliosis |
| 39 | F | 26 | c.7552+4-7552+7del | － | Splicing | VUS | I50 | Novel | ＋ |  | 2 | II | III | － |
| 40 | F | 50 | c.7752+4_7752+7del | － | Splicing | VUS | I50 | Novel | ＋ |  | 2 | － | III | － |
| 41 | M | 7 | c.1260+1_1260+2insT | － | Splicing | LP | I11 | Novel | － |  | 2 | II | I | Scaphoid developmental abnormalities, Learning disabilities |
| 42 | F | 1 | c.654+1G>C | － | Splicing | P | I6 | Novel | － |  | 1 | II | － | － |
| 43 | F | 32 | c.6859-6T>A | － | Splicing | VUS | I45 | Novel | ＋ | Maternal | 2 | II | II | － |
| 44 | M | 27 | c.6859-6T>A | － | Splicing | VUS | I45 | Novel | ＋ | Maternal | 2 | II | II | － |
| 45 | F | 55 | c.6859-6T>A | － | Splicing | VUS | I45 | Novel | ＋ |  | 2 | II | III | － |
| 46 | F | 4 | c.3365C>T | p.(Gln1189Ter) | Nonsense | LP | E27 | Reported | ＋ | paternal | 1 | II | － | Halo-like phenomenon on Dermal melanocytosis |
| 47 | F | 6 | c.4892T>A | p.(Leu1610Ter) | Nonsense | P | E36 | Reported | － |  | 1 | III | － | Axillary and/or inguinal freckles, AN |
| 48 | F | 26 | c.569T>A | p.(Leu190Ter) | Nonsense | P | E5 | Reported | － |  | 2 | III | I | Axillary and/or inguinal freckles, Scoliosis, Learning disabilities, AN |
| 49 | M | 2 | c.2180C>A | p.(Ser727Ter) | Nonsense | P | E18 | Reported | ＋ |  | 2 | II | － | OPG |
| 50 | F | 2 | c.681T>G | p.(Tyr227Ter) | Nonsense | P | E7 | Reported | － | mosaic | 2 | I | － | Funnel chest, AN, Halo-like phenomenon on Dermal melanocytosis, JXGs |
| 51 | M | 2 | c.2125T>C | p.(Cys709Arg) | Missense | LP | E18 | Reported | － |  | 1 | III | II | AN, Halo-like phenomenon on Dermal melanocytosis |
| 52 | F | 33 | c.6085G>T | p.(Val2029Phe) | Missense | LP | E42 | Reported | － |  | 2 | III | III | Axillary and/or inguinal freckles, JXGs |
| 53 | F | 10 | c.6881T>G | p.(Leu2294Arg) | Missense | LP | E47 | Reported | － |  | 5 | II | IV | PN, Scoliosis, Learning disabilities |
| 54 | M | 1 | c.3544G>T | p.(Val1182Gly) | Missense | LP | E27 | Reported | － |  | 1 | II | － | － |
| 55 | M | 29 | c.1229T>C | p.(Leu410Pro) | Missense | P | E11 | Reported | － |  | 2 | II | III | AN, JXGs |
| 56 | F | 0 | c.2900T>G | p.(Ile967Arg) | Missense | VUS | E22 | Reported | － |  | 1 | II | － | AN, Halo-like phenomenon on Dermal melanocytosis |
| 57 | F | 3 | c.5788T>C | p.(Cys1930Arg) | Missense | P | E39 | Reported | － |  | 1 | II | I | AN, JXGs |
| 58 | F | 55 | c.7258G>A | p.(Val2420Ile) | Missense | VUS | E48 | Reported | － |  | 2 | II | III | － |
| 59 | M | 6 | c.3456_3459delACTC | p.(Leu1152LeufsTer5) | Frameshift | P | E26 | Reported | － |  | 2 | II | － | Axillary and/or inguinal freckles, Bony abnormalities, Halo-like phenomenon on Dermal melanocytosis |
| 60 | M | 38 | c.3619delA | p.(Arg1207AspfsTer8) | Frameshift | P | E27 | Reported | ＋ |  | 2 | I | III | Axillary and/or inguinal freckles, Scoliosis, Learning disabilities |
| 61 | F | 2 | c.5587_5591delTTTAA | p.(Phe1863PhefsTer27) | Frameshift | P | E38 | Reported | － |  | 1 | I | － | Axillary and/or inguinal freckles, AN |
| 62 | M | 11 | c.7201delA | p.(Lys2401AsnfsTer10) | Frameshift | P | E48 | Reported | － |  | 1 | II | － | － |
| 63 | M | 8 | c.5667dupT | p.(Ile1890TyrfsTer2) | Frameshift | P | E38 | Reported | ＋ |  | 5 | II | I | PN, Axillary and/or inguinal freckles, Lisch nodules |
| 64 | F | 7 | c.4368-1G>A | － | Splicing | P | I33 | Reported | － |  | 5 | II | I | PN, Scoliosis, Learning disabilities, JXGs |
| 65 | M | 0 | c.7000-1G>T | － | Splicing | P | I46 | Reported | － |  | 1 | I | － | Axillary and/or inguinal freckles |
| 66 | F | 32 | c.7000-1G>A | － | Splicing | LP | I45 | Reported | ＋ |  | 2 | II | III | Axillary and/or inguinal freckles |
| 67 | M | 1 | c.1198C>T | p.(Gln400Ter) | Nonsense | P | E11 | Reported | ＋ |  | 1 | II | － | Halo-like phenomenon on Dermal melanocytosis |
| 68 | M | 13 | c.1660C>T | p.(Gln554Ter) | Nonsense | LP | E15 | Reported | － | mosaic | 1 | II | － | Axillary and/or inguinal freckles, AN |
| 69 | M | 9 | c.3943C>T | p.(Gln1315Ter) | Nonsense | P | E29 | Reported | － |  | 2 | II | II | Axillary and/or inguinal freckles, Learning disabilities, AN |
| 70 | M | 4 | c.4107C>G | p.(Tyr1369Ter) | Nonsense | P | E30 | Reported | － |  | 1 | III | － | Axillary and/or inguinal freckles, Learning disabilities, JXGs |
| 71 | F | 2 | c.6619C>T | p.(Gln2207Ter) | Nonsense | P | E44 | Reported | － |  | 1 | II | I | Axillary and/or inguinal freckles, Halo-like phenomenon on Dermal melanocytosis, JXGs |
| 72 | F | 3 | c.943C>T | p.(Gln315Ter) | Nonsense | P | E9 | Reported | － |  | 2 | II | － | Axillary and/or inguinal freckles, Unequal length of legs, AN |
| 73 | F | 9 | c.5839C>T | p.(Arg1947Ter) | Nonsense | P | E39 | Reported | － |  | 1 | II | － | Axillary and/or inguinal freckles, Learning disabilities, AN |
| 74 | M | 2 | c.1238C>G | p.(Ser413Ter) | Nonsense | P | E11 | Reported | － |  | 1 | II | － | － |
| 75 | F | 2 | c.1246C>T | p.(Arg416Ter) | Nonsense | P | E11 | Reported | － |  | 2 | II | I | Axillary and/or inguinal freckles, Tibial degeneration |
| 76 | M | 4 | c.1246C>T | p.(Arg416Ter) | Nonsense | P | E11 | Reported | － |  | 5 | II | I | PN, AN, Halo-like phenomenon on Dermal melanocytosis |
| 77 | F | 8 | c.1318C>T | p.(Arg440Ter) | Nonsense | P | E12 | Reported | － |  | 1 | II | I | Learning disabilities |
| 78 | F | 34 | c.1318C>T | p.(Arg440Ter) | Nonsense | P | E12 | Reported | － |  | 1 | III | II | Axillary and/or inguinal freckles, JXGs |
| 79 | F | 4 | c.3449C>G | p.(Ser1150Ter) | Nonsense | P | E26 | Reported | － |  | 1 | II | － | － |
| 80 | F | 25 | c.4265C>G | p.(Ser1422Ter) | Nonsense | P | E31 | Reported | － |  | 2 | II | II | Axillary and/or inguinal freckles, JXGs |
| 81 | M | 19 | c.4537C>T | p.(Arg1513Ter) | Nonsense | P | E34 | Reported | － |  | 2 | II | III | Axillary and/or inguinal freckles |
| 82 | M | 8 | c.4537C>T | p.(Arg1513Ter) | Nonsense | P | E34 | Reported | － |  | 2 | II | II | Lisch nodules, Calf deformity, Learning disabilities, AN |
| 83 | F | 0 | c.5722G>T | p.(Glu1908Ter) | Nonsense | LP | E38 | Reported | － |  | 1 | II | － | Halo-like phenomenon on Dermal melanocytosis |
| 84 | M | 1 | c.5839C>T | p.(Arg1947Ter) | Nonsense | P | E39 | Reported | － |  | 1 | II | － | AN, Halo-like phenomenon on Dermal melanocytosis |
| 85 | F | 4 | c.6841C>T | p.(Gln2281Ter) | Nonsense | P | E45 | Reported | － |  | 1 | I | － | － |
| 86 | F | 5 | c.910C>T | p.(Arg304Ter) | Nonsense | P | E9 | Reported | ＋ | Maternal | 1 | II | I | Axillary and/or inguinal freckles |
| 87 | M | 11 | c.1246C>T | p.(Arg416Ter) | Nonsense | P | E11 | Reported | ＋ |  | 2 | III | III | Axillary and/or inguinal freckles, Scoliosis, AN, Halo-like phenomenon on Dermal melanocytosis |
| 88 | M | 60 | c.1275G>A | p.(Trp425Ter) | Nonsense | P | E12 | Reported | － |  | 2 | II | III | － |
| 89 | M | 6 | c.1318C>T | p.(Arg440Ter) | Nonsense | P | E12 | Reported | ＋ | Maternal | 1 | I | － | － |
| 90 | F | 30 | c.1318C>T | p.(Arg440Ter) | Nonsense | P | E12 | Reported | － |  | 1 | IV | I | Axillary and/or inguinal freckles |
| 91 | F | 56 | c.1381C>T | p.(Arg461Ter) | Nonsense | P | E12 | Reported | － |  | 2 | II | IV | Axillary and/or inguinal freckles |
| 92 | M | 3 | c.1381C>T | p.(Arg461Ter) | Nonsense | P | E12 | Reported | － | Maternal | 1 | II | － | － |
| 93 | M | 34 | c.247C>T | p.(Gln83Ter) | Nonsense | P | E3 | Reported | ＋ |  | 5 | II | II | PN, Axillary and/or inguinal freckles, Mediastinal tumor, Halo-like phenomenon on Dermal melanocytosis |
| 94 | F | 0 | c.3163C>T | p.(Gln1055Ter) | Nonsense | P | E4 | Reported | － |  | 1 | II | － | － |
| 95 | M | 22 | c.3520C>T | p.(Gln1174Ter) | Nonsense | P | E27 | Reported | － |  | 1 | II | － | － |
| 96 | M | 31 | c.3814C>T | p.(Gln1272Ter) | Nonsense | P | E28 | Reported | － |  | 1 | II | － | － |
| 97 | M | 6 | c.3826C>T | p.(Arg1276Ter) | Nonsense | P | E28 | Reported | － |  | 1 | II | － | Halo-like phenomenon on Dermal melanocytosis |
| 98 | F | 23 | c.3826C>T | p.(Arg1276Ter) | Nonsense | P | E28 | Reported | － |  | 1 | III | － | Axillary and/or inguinal freckles |
| 99 | F | 14 | c.4108C>T | p.(Gln1370Ter) | Nonsense | P | E30 | Reported | － |  | 5 | II | I | Retroperitoneal junctional tumor, Tibial flexion |
| 100 | F | 20 | c.4265C>G | p.(Ser1422Ter) | Nonsense | LP | E32 | Reported | ＋ |  | 1 | II | I | － |
| 101 | F | 35 | c.4537C>T | p.(Arg1513Ter) | Nonsense | P | E34 | Reported | － |  | 2 | II | I | Axillary and/or inguinal freckles, Scoliosis |
| 102 | F | 34 | c.4537C>T | p.(Arg1513Ter) | Nonsense | P | E34 | Reported | － |  | 2 | III | II | － |
| 103 | M | 46 | c.484C>T | p.(Gln162Ter) | Nonsense | P | E5 | Reported | － |  | 2 | II | III | － |
| 104 | F | 39 | c.5242C>T | p.(Arg1748Ter) | Nonsense | P | E37 | Reported | － |  | 1 | II | II | － |
| 105 | F | 0 | c.5242C>T | p.(Arg1748Ter) | Nonsense | P | E37 | Reported | － |  | 1 | II | － | － |
| 106 | F | 2 | c.541C>T | p.(Gln181Ter) | Nonsense | P | E5 | Reported | － |  | 2 | I | － | Axillary and/or inguinal freckles, Scoliosis, AN |
| 107 | F | 38 | c.574C>T | p.(Arg192Ter) | Nonsense | P | E5 | Reported | ＋ |  | 2 | III | III | Axillary and/or inguinal freckles, Learning disabilities, AN, Halo-like phenomenon on Dermal melanocytosis, JXGs |
| 108 | F | 17 | c.5839C>T | p.(Arg1947Ter) | Nonsense | P | E39 | Reported | － |  | 5 | II | III | PN, Axillary and/or inguinal freckles, Learning disabilities |
| 109 | F | 11 | c.6709C>T | p.(Arg2237Ter) | Nonsense | P | E45 | Reported | － |  | 5 | II | － | PN, Axillary and/or inguinal freckles, Learning disabilities, Scoliosis |
| 110 | F | 14 | c.6709C>T | p.(Arg2237Ter) | Nonsense | P | E45 | Reported | － |  | 2 | II | III | － |
| 111 | M | 25 | c.6791dup | p.(Tyr2264Ter) | Frameshift | P | E46 | Reported | ＋ | Maternal | 2 | II | II | Learning disabilities, Halo-like phenomenon on Dermal melanocytosis |
| 112 | M | 28 | c.6792C>A | p.(Tyr2264Ter) | Nonsense | P | E45 | Reported | － | paternal | 5 | II | II | PN, Axillary and/or inguinal freckles, Learning disabilities, AN |
| 113 | M | 4 | c.7681C>T | p.(Gln2561Ter) | Nonsense | P | E52 | Reported | － |  | 1 | II | － | － |
| 114 | F | 68 | c.7846C>T | p.(Arg2616Ter) | Nonsense | P | E53 | Reported | － |  | 2 | － | IV | － |
| 115 | F | 47 | c.7846C>T | p.(Arg2616Ter) | Nonsense | P | E53 | Reported | ＋ |  | 2 | － | III | － |
| 116 | M | 6 | c.271G>T | p.(Glu91Ter) | Nonsense | LP | E3 | Reported | － |  | 2 | I | － | Epilepsy, Learning disabilities |
| 117 | F | 4 | c.2072T>G | p.(Leu691Arg) | Missense | LP | E18 | Reported | － |  | 1 | II | － | Axillary and/or inguinal freckles |
| 118 | F | 6 | c.2342A>C | p.(Gln781Pro) | Missense | LP | E20 | Reported | － |  | 1 | I | I | Axillary and/or inguinal freckles, AN, Halo-like phenomenon on Dermal melanocytosis |
| 119 | M | 2 | c.4367G>C | p.(Arg1456Thr) | Missense | VUS | E33 | Reported | － |  | 1 | II | － | Axillary and/or inguinal freckles |
| 120 | M | 5 | c.1466A>G | p.(Tyr489Cys) | Missense | P | E13 | Reported | － |  | 1 | III | I | Axillary and/or inguinal freckles |
| 121 | F | 1 | c.1748A>G | p.(Lys583Arg) | Missense | P | E16 | Reported | － |  | 1 | II | － | Axillary and/or inguinal freckles |
| 122 | M | 4 | c.1885G>A | p.(Gly629Arg) | Missense | P | E17 | Reported | － |  | 1 | III | － | － |
| 123 | M | 1 | c.2537C>A | p.(Ala846Asp) | Missense | P | E21 | Reported | － |  | 1 | II | － | Halo-like phenomenon on Dermal melanocytosis |
| 124 | M | 20 | c.2540T>C | p.(Leu847Pro) | Missense | P | E21 | Reported | － |  | 1 | II | I | Axillary and/or inguinal freckles, Halo-like phenomenon on Dermal melanocytosis |
| 125 | F | 1 | c.2540T>C | p.(Leu847Pro) | Missense | P | E21 | Reported | － |  | 1 | II | － | Axillary and/or inguinal freckles |
| 126 | M | 1 | c.2T>C | p.(Met1Thr) | Missense | P | E1 | Reported | － |  | 1 | II | － | JXGs |
| 127 | F | 2 | c.4168C>T | p.(Leu1390Phe) | Missense | P | E31 | Reported | － |  | 2 | II | － | Tibial pseudarthrosis |
| 128 | M | 36 | c.1229_1230delTGinsCT | p.(Leu410Pro) | Missense | P | E11 | Reported | ＋ |  | 2 | II | II | － |
| 129 | F | 14 | c.1466A>G | p.(Tyr489Cys) | Missense | P | E13 | Reported | － |  | 1 | II | I | － |
| 130 | M | 2 | c.2350T>C | p.(Trp784Arg) | Missense | LP | E20 | Reported | － |  | 1 | III | － | Axillary and/or inguinal freckles, AN |
| 131 | F | 1 | c.2530C>T | p.(Leu844Phe) | Missense | P | E21 | Reported | － |  | 2 | I | － | Scoliosis |
| 132 | M | 6 | c.277T>C | p.(Cys93Arg) | Missense | LP | E3 | Reported | － |  | 1 | － | I | － |
| 133 | F | 10 | c.3569G>A | p.(Gly1190Asp) | Missense | LP | E27 | Reported | － |  | 1 | II | － | Axillary and/or inguinal freckles |
| 134 | M | 0 | c.3644T>A | p.(Met1215Lys) | Missense | VUS | E27 | Reported | － |  | 1 | II | － | Axillary and/or inguinal freckles, AN, JXGs |
| 135 | F | 24 | c.3827G>A | p.(Arg1276Gln) | Missense | P | E28 | Reported | － |  | 1 | I | I | － |
| 136 | F | 10 | c.4981T>C | p.(Cys1661Thr) | Missense | LP | E37 | Reported | ＋ | paternal | 1 | II | － | － |
| 137 | M | 1 | c.5489C>T | p.(Pro1830Leu) | Missense | LP | E37 | Reported | － |  | 1 | II | － | － |
| 138 | F | 24 | c.5546G>T | p.(Arg1849Leu) | Missense | LP | E37 | Reported | ＋ |  | 1 | II | II | Axillary and/or inguinal freckles, JXGs |
| 139 | M | 1 | c.6858G>C | p.(Lys2286Asn) | Missense | VUS | E45 | Reported | － |  | 1 | I | － | － |
| 140 | M | 1 | c.6858G>C | p.(Lys2286Asn) | Missense | VUS | E45 | Reported | － |  | 1 | II | － | － |
| 141 | F | 15 | c.968C>A | p.(Ala323Asp) | Missense | P | E9 | Reported | ＋ |  | 2 | II | I | － |
| 142 | M | 12 | c.6540delC | p.(Ser2181ValfsTer19) | Frameshift | P | E43 | Reported | － |  | 1 | II | I | Axillary and/or inguinal freckles, Learning disabilities, JXGs |
| 143 | F | 3 | c.2970_2971del | p.(Thr991fs) | Frameshift | P | E22 | Reported | － |  | 1 | II | － | － |
| 144 | M | 0 | c.1541_1542delAG | p.(Gln514ArgfsTer43) | Frameshift | P | E14 | Reported | － |  | 1 | II | － | － |
| 145 | F | 1 | c.1753dupT | p.(Leu585PhefsTer3) | Frameshift | P | E16 | Reported | － |  | 1 | II | － | Axillary and/or inguinal freckles, Halo-like phenomenon on Dermal melanocytosis, JXGs |
| 146 | F | 1 | c.1754_1757del | p.(Thr586ValfsTer18) | Frameshift | P | E16 | Reported | － |  | 1 | II | － | Axillary and/or inguinal freckles, AN |
| 147 | F | 1 | c.5046dupT | p.(Ile1803TyrfsTer38) | Frameshift | P | E37 | Reported | － |  | 1 | II | － | AN |
| 148 | F | 23 | c.1541_1542delAG | p.(Gln514ArgfsTer43) | Frameshift | P | E14 | Reported | － |  | 5 | II | II | PN, Axillary and/or inguinal freckles |
| 149 | M | 1 | c.1679delT | p.(Leu560CysfsTer8) | Frameshift | P | E15 | Reported | － |  | 1 | II | － | － |
| 150 | F | 25 | c.2033_2034insC | p.(Ile679AspfsTer21) | Frameshift | P | E18 | Reported | ＋ |  | 2 | I | III | Axillary and/or inguinal freckles |
| 151 | M | 22 | c.3457_3460delCTCA | p.(Leu1153MetfsTer4) | Frameshift | P | E26 | Reported | － |  | 2 | II | II | PN |
| 152 | F | 24 | c.3947_3948delAT | p.(His1316ArgfsTer2) | Frameshift | P | E29 | Reported | － |  | 2 | III | II | Axillary and/or inguinal freckles, Learning disabilities, JXGs |
| 153 | F | 0 | c.7192_7193delGT | p.(Leu2398GlyfsTer2) | Frameshift | P | E48 | Reported | － |  | 1 | II | － | Axillary and/or inguinal freckles, AN, Halo-like phenomenon on Dermal melanocytosis |
| 154 | F | 28 | c.3457_3460delACTC | p.(Leu1153MetfsTer4) | Frameshift | P | E26 | Reported | － |  | 5 | II | II | PN, Axillary and/or inguinal freckles |
| 155 | M | 2 | del | － | Exon deletion | P | E1~58 | Reported | － |  | 1 | II | － | Learning disabilities, AN, Halo-like phenomenon on Dermal melanocytosis |
| 156 | F | 29 | del | － | Exon deletion | P | E1~58 | Reported | － |  | 1 | III | I | Axillary and/or inguinal freckles |
| 157 | F | 15 | del | － | Exon deletion | P | E1~58 | Reported | － |  | 1 | II | － | － |
| 158 | M | 20 | del | － | Exon deletion | P | E1~58 | Reported | － |  | 1 | III | II | Axillary and/or inguinal freckles |
| 159 | F | 13 | del | － | Exon deletion | P | E1~58 | Reported | － |  | 1 | II | － | － |
| 160 | F | 30 | del | － | Exon deletion | P | E1~58 | Reported | － |  | 2 | II | III | － |
| 161 | F | 5 | del | － | Exon deletion | P | E1~57 | Reported | － |  | 2 | II | I | PN, Axillary and/or inguinal freckles |
| 162 | F | 0 | del | － | Exon deletion | P | E1~58 | Reported | － |  | 1 | I | － | － |
| 163 | M | 3 | del | － | Exon deletion | P | E1~58 | Reported | － |  | 1 | I | － | Axillary and/or inguinal freckles, Tibial pseudarthrosis |
| 164 | M | 7 | del | － | Exon deletion | P | E1~58 | Reported | － |  | 2 | II | I | Axillary and/or inguinal freckles, Chicken breast, Learning disabilities, Halo-like phenomenon on Dermal melanocytosis, JXGs |
| 165 | F | 27 | del | － | Exon deletion | P | E1~58 | Reported | － |  | 2 | III | II | Axillary and/or inguinal freckles, Halo-like phenomenon on Dermal melanocytosis |
| 166 | F | 32 | del | － | Exon deletion | P | E1~58 | Reported | － |  | 1 | III | I | PN, Axillary and/or inguinal freckles, bony abnormalities, Learning disabilities, Halo-like phenomenon on Dermal melanocytosis, JXGs |
| 167 | M | 26 | del | － | Exon deletion | P | E1~58 | Reported | － |  | 1 | II | － | － |
| 168 | F | 9 | del | － | Exon deletion | P | E1~58 | Reported | － |  | 1 | II | I | － |
| 169 | M | 10 | del | － | Exon deletion | P | E1~58 | Reported | － |  | 2 | II | IV | PN, Lisch nodules, Scoliosis, Halo-like phenomenon on Dermal melanocytosis, JXGs |
| 170 | F | 8 | del | － | Exon deletion | P | E2~58 | Reported | － |  | 2 | III | I | PN, Axillary and/or inguinal freckles |
| 171 | F | 26 | del | － | Exon deletion | P | E1~58 | Reported | － |  | 2 | III | II | Axillary and/or inguinal freckles |
| 172 | M | 23 | del | － | Exon deletion | P | E1~58 | Reported | － |  | 2 | II | III | － |
| 173 | M | 19 | del | － | Exon deletion | P | E35 | Reported | － |  | 2 | II | III | － |
| 174 | M | 4 | del | － | Exon deletion | P | E9~11 | Reported | － |  | 5 | II | I | PN, Axillary and/or inguinal freckles, Halo-like phenomenon on Dermal melanocytosis |
| 175 | M | 1 | c.1185+1G>A | － | Splicing | P | I10 | Reported | ＋ |  | 2 | II | － | Axillary and/or inguinal freckles, Rib exostosis, AN, Halo-like phenomenon on Dermal melanocytosis |
| 176 | M | 30 | c.480-1G>A | － | Splicing | P | I4 | Reported | － |  | 2 | III | II | Axillary and/or inguinal freckles |
| 177 | M | 5 | c.2409+1G>A | － | Splicing | P | I20 | Reported | － |  | 1 | II | I | Axillary and/or inguinal freckles |
| 178 | M | 5 | c.3113+1G>A | － | Splicing | P | I23 | Reported | － |  | 2 | II | － | Axillary and/or inguinal freckles, Epilepsy |
| 179 | F | 10 | c.4515-1G>A | － | Splicing | P | I33 | Reported | － |  | 1 | III | － | Axillary and/or inguinal freckles |
| 180 | F | 1 | c.4773-2A>C | － | Splicing | P | I33 | Reported | － |  | 1 | II | － | － |
| 181 | F | 0 | c.7000-2A>G | － | Splicing | P | I45 | Reported | － |  | 2 | II | － | Tibial pseudarthrosis, AN |
| 182 | F | 5 | c.7000-2A>G | － | Splicing | P | I45 | Reported | － |  | 1 | II | I | Halo-like phenomenon on Dermal melanocytosis |
| 183 | M | 2 | c.2410-16A>G | － | Splicing | P | I20 | Reported | － |  | 1 | II | － | － |
| 184 | M | 2 | c.2410-18C>G | － | Splicing | LP | I20 | Reported | － |  | 1 | II | － | Axillary and/or inguinal freckles, AN, Halo-like phenomenon on Dermal melanocytosis |
| 185 | F | 56 | c.3871-1G>T | － | Splicing | P | I28 | Reported | － |  | 1 | III | I | Axillary and/or inguinal freckles |
| 186 | M | 0 | c.4773-2A>C | － | Splicing | P | I36 | Reported | － |  | 1 | II | － | － |
| 187 | F | 16 | c.6756+1G>T | － | Splicing | P | I44 | Reported | － |  | 1 | IV | I | Axillary and/or inguinal freckles |
| 188 | F | 24 | c.6756+2T>C | － | Splicing | P | I44 | Reported | ＋ |  | 1 | III | I | Axillary and/or inguinal freckles |
| 189 | M | 0 | dup | － | Exon duplication | P | E2~27 | Reported | ＋ | Maternal | 1 | I | － | － |
| 190 | M | 27 | c.1185G>A | p.(Lys395Lys) | Synonymous | LP | E10 | Reported | ＋ |  | 5 | III | II | PN, Axillary and/or inguinal freckles |
| 191 | M | 25 | c.7089_7094delTAACTT | p.(Ser2384_Phe2386delinsSer) | Indel | LP | E47 | Reported | ＋ | Maternal | 2 | I | II | Axillary and/or inguinal freckles, JXGs |
| 192 | F | 7 | c.3639_3641delAAT | p.(Thr1213_Met1214delinsThr) | Indel | P | E27 | Reported | ＋ | paternal | 1 | II | － | Axillary and/or inguinal freckles, Learning disabilities |
| 193 | F | 23 | c.3639_3641del | p.(Met1215del) | Indel | P | E27 | Reported | ＋ | paternal | 1 | II | I | － |

This table includes 193 patients with detected NF1 variants, including 182 patients with pathogenic/likely pathogenic variants and 11 patients with variants of uncertain significance. Eighteen patients without reportable NF1 variants were not included.

P: pathogenic; LP: likely pathogenic; VUS: variant of uncertain significance; E: indicates exon; I: indicates intron; CALMs, café-au-lait macules; PN, plexiform neurofibroma; AN, nevus anemicus; JXG, juvenile xanthogranuloma; OPG, optic pathway glioma. ＋ indicates positive family history; － indicates no family history.

I: 1-9; II: 10-99; III: 100-999; IV:>1000
