# Supplementary material for: Clinical characteristics and variant spectrum of NF1 in Chinese patients with neurofibromatosis type 1: a cross-sectional study
Source: Orphanet J Rare Dis. 2026 Aug 17;21:416. doi: 10.1186/s13023-026-04534-9 (PMC13756884; doi:10.1186/s13023-026-04534-9)
Supplement: Supplementary file 7 [file 13023_2026_4534_MOESM8_ESM.docx]

**Supplement 8. Exploratory multivariable logistic regression analysis of factors associated with DNB-defined severe classification among patients with detected *NF1* variants**

| **Variables** | **β** | **Odds ratio** | **95%*CI*** |
| --- | --- | --- | --- |
| **Gender** |  |  |  |
| Male vs. Female | -0.022 | 0.978 | 0.268~3.578 |
| Female |  | REF |  |
| **Age** |  |  |  |
| ≤9 vs. >25 | -0.844 | 0.430 | 0.071~2.592 |
| 10-25 vs. >25 | -1.104 | 0.332 | 0.078~1.411 |
| >25 |  | REF |  |
| **Family history** |  |  |  |
| Yes |  | REF |  |
| No vs. Yes | -0.865 | 0.421 | 0.106~1.668 |
| **Maximum diameter of CALMs (cm)** |  |  |  |
| ≤5 |  | REF |  |
| 6~8 vs. ≤5 | 0.548 | 1.729 | 0.429~6.966 |
| >8 vs. ≤5 | 0.131 | 1.140 | 0.295~4.406 |
| **Quantity of CALMs** |  |  |  |
| 0~9 |  | REF |  |
| 10~99 vs. 0~9 | 2.613 | 13.640* | 1.018~182.698 |
| >99 vs. 0~9 | 1.825 | 6.203 | 0.397~96.871 |
| **Quantity of neurofibromas** |  |  |  |
| 0 |  | REF |  |
| 1~9 vs. 0 | -2.266 | 0.104* | 0.017~0.632 |
| 10~99 vs. 0 | -0.656 | 0.519 | 0.087~3.110 |
| >99 vs. 0 | - | - | - |
| **Learning disabilities** |  |  |  |
| Yes |  | REF |  |
| No vs. Yes | -0.828 | 0.437 | 0.089~2.146 |
| **AN** |  |  |  |
| Yes |  | REF |  |
| No vs. Yes | -1.169 | 0.311 | 0.048~2.005 |
| **Halo-like phenomenon on dermal melanocytosis** |  |  |  |
| Yes |  | REF |  |
| No vs. Yes | 0.203 | 1.225 | 0.271~5.530 |
| **Age of onset** |  |  |  |
| 0 vs. ≥2 | -1.129 | 0.323 | 0.008~13.004 |
| >0-2 vs. ≥2 | -0.126 | 0.881 | 0.038~20.640 |
| ≥2 |  | REF |  |
| **Age of onset of CALMs** |  |  |  |
| 0 vs. >4 | 0.473 | 1.605 | 0.025~101.957 |
| 1-4 vs. >4 | -0.653 | 0.520 | 0.019~14.545 |
| >4 |  | REF |  |
| **Age of onset of neurofibromas** |  |  |  |
| ≤4 vs. >11 | 0.009 | 1.009 | 0.234~4.358 |
| 5-11 vs. >11 | 0.381 | 1.463 | 0.324~6.601 |
| >11 |  | REF |  |
| **Alterations in neurofibromin structure** |  |  |  |
| Small vs. Big | 1.302 | 3.677 | 0.625~21.643 |
| Big |  | REF |  |
| **VDs** |  |  |  |
| VD1 |  | REF |  |
| VD2–5/overlapping category vs. VD1 | -0.452 | 0.637 | 0.165~2.462 |

REF: reference group

OR<1 indicates lower odds of DNB-defined severe classification.

The category “>99 neurofibromas” was not estimated separately because of model instability.

Given overlap between several clinical features and DNB staging, results are presented as cross-sectional associations rather than independent prognostic predictors. Small subgroup sizes may contribute to wide confidence intervals and model instability.

**P*<0.05; ***P*<0.01.
